# [(7-chloroquinolin-4-yl)amino]acetophenones and their copper(II) derivatives: Synthesis, characterization, computational studies and antimalarial activity

**DOI:** 10.17179/excli2019-1805

**Published:** 2019-10-28

**Authors:** Yonathan de J. Parra, Felix D. Andueza L, Rosa E. Ferrer M, Julia Bruno Colmenarez, María E. Acosta, Jaime Charris, Philip J. Rosenthal, Jiri Gut

**Affiliations:** 1 Escuela de Ingeniería Ambiental, Facultad de Ingeniería en Geología, Minas, Petróleos y Ambiental, Universidad Central del Ecuador, Quito 170521, Ecuador; 2 Departamento de Química, Facultad de Humanidades y Educación, Universidad del Zulia, Apartado 526, Maracaibo, Estado Zulia, Venezuela; 3 Centro de Investigación y Tecnología de Materiales (CITeMa), Instituto Venezolano de Investigaciones Científicas, Apartado 20632, Altos de Pipe 1020-A Estado Miranda, Venezuela; 4 Laboratorio de Bioquímica, Facultad de Farmacia, Universidad Central de Venezuela, Apartado 47206, Los Chaguaramos, 1041-A Caracas, Venezuela; 5 Laboratorio de Síntesis Orgánica, Facultad de Farmacia, Universidad Central de Venezuela, Apartado 47206, Los Chaguaramos, 1041-A Caracas, Venezuela; 6 Department of Medicine, University of California, Box 0811, San Francisco, California 94143, USA

**Keywords:** acetophenone, antimalarial, copper, DFT, fragmentation, quinoline

## Abstract

The synthesis of the compounds [(7-chloroquinolin-4-yl)amino]acetophenones (**4**, **5**) and their copper(II) complexes (**4a**, **5a**) is reported. The compounds were characterized using a wide range of spectroscopic and spectrometric techniques, such as FTIR, UV-vis, NMR, EPR, ESI-CID-MS^2^. The spectral results suggested that the ligand acted as chelating species coordinating the metal through the endocyclic nitrogen of the quinoline ring in both complexes, with general formulae expressed in two ways, according to the phase in which they are: [Cu(L)_2_Cl_2_] for solid phase and [Cu(L)_2_][2Cl] for liquid phase. The EPR study of the Cu (II) complexes indicated a probable distorted tetrahedral coordination geometry. This result was confirmed by the calculated optimized structures at the DFT/B3LYP method with the 6-31G (d,p) basis set. The characterization of the fragmentation pattern of protonated free ligands was extended here to fragments as low as *m/z* 43, while for coordination complexes it extends to fragments at *m/z* 80 and *m/z* 111. The antimalarial activity of the compounds was determined through three different tests: inhibitory activity against *in vitro* growth of *Plasmodium falciparum* (W2), inhibition of hemozoin formation (β-hematin) and *in vitro* inhibitory activity against recombinant falcipain-2, where compound 5 showed considerable activity. However, the activity of free ligands against *P. falciparum* was increased by complexing with the Cu (II) metal ion. The values of the HOMO-LUMO energy gap of 3.847 eV (**4a**) and 3.932 eV (**5a**) were interpreted with high chemical activity and thus, could influence on biological activity. In both compounds, the total electron density surface mapped with electrostatic potential clearly revealed the presence of high negative charge on the Cu atom. Also, this study reported the molecular docking of free ligands (**4**, **5**) using software package ArgusLab 4.0.1. The results revealed the importance of water molecules as interaction bridges through hydrogen bonds between free ligands and β-hematin; at the same time, the hypothesis that π-π interaction between quinoline derivatives and the electronic system of hematin governs the formation of adducts was confirmed.

## Introduction

Malaria, a traditional infectious disease, added 219 million new malaria cases worldwide by 2017, with an estimated 435,000 deaths of which approximately 61 % were children under five years (WHO, 2018[47]). There are therapeutic treatments that belong to the 4-aminoquinolines family that reduce the mortality rate for malaria. However, many medications related to this family have decreased in effectiveness given the ability of Plasmodium (mainly falciparum, vivax and malariae) to develop resistance, a major obstacle in the fight against malaria (Parra and Ferrer, 2012[30]).

These facts lead to the need of development of new drugs to reduce resistance events and increasing the effectiveness of such substances; for this, various drug design methodologies have been proposed, including the synthesis of molecules based on leading compounds or pharmacophores, molecular hybridization and metal complexing (Kouznetsov and Amado, 2008[21]; Kouznetsov and Gómez-Barrio, 2009[22]; Camacho et al., 2005[4]).

In this sense, as part of our research on the synthesis and biological evaluation of 4-aminoquinolines with potential antimalarial activity, we report here synthetic, spectroscopic, spectrometric, computational aspects and antimalarial activity of compounds based on the pharmacophore nucleus 7-chloro-4-aminoquinoline (antimalarial) (Egan et al., 2000[10]), acetophenone unit to supply other reactive sites (electrophilic unit) (Chiyanzu et al., 2005[6]) and metallic centers of kind Cu(II) to potentiate pharmacological properties, (Bahl et al., 2010[3]) through possible participation in the metabolic distribution of copper in *Plasmodium falciparum* (Rasoloson et al., 2004[34]), potential mechanism for new pathways of antimalarial drugs.

## Materials and Methods

All reagents and solvents used were of analytical grade. Organic chemicals and metal salts, such as 4,7-dichloro-quinoline, aminoacetophenones and cupric chloride were obtained from Aldrich Chemical Co, USA.

### General procedures

The solvents were subjected to previous purification and drying processes by standard methods. The progress of the reactions and purity of the synthesized compounds was evaluated by thin-layer chromatography (TLC) using silica gel 60 plates with UV254 fluorescent indicator, an ultraviolet lamp and a suitable solvent system.

Melting points were measured with a Stuart™ SMP3 melting point apparatus (Sigma-Aldrich). Infrared spectra were recorded on KBr pellets with a Shimadzu FTIR 8400 spectrophotometer (400-4000 cm^-1^). Nuclear Magnetic Resonance spectra were taken using two spectrometers, one of Jeol Eclipse brand and another Bruker Avance II brand, of 270 MHz and 300 MHz for ^1^H NMR, respectively. Both devices have the power of 67.9 MHz for ^13^C NMR.

Electron Paramagnetic Resonance (EPR) spectra were taken at 24 °C on a reconstructed and automated X-band Varian E-Line spectrometer. A rectangular cavity resonating in the TE-102 mode was used, with modulation of 100 kHz, using a microwave power of 5 mW. ESI-CID-MS^2^ spectra were recorded using a TSQ Quantum triple-quadrupole mass spectrometer (Thermo Scientific brand) coupled to a liquid chromatography (CL) system.

### Synthesis of free ligands (4 and 5)

The synthesis of the free ligands was carried out applying the protocol of Ferrer et al. (2009[11]). In a two-necked reaction flask, a mixture was prepared in a molar ratio 1:1 of the reactants, by dissolving 0.5 g (2.5 mmol) of 4,7-dichloro-quinoline (**1**) in dry ethanol to then add 0.37 g (2.75 mmol) of the 4- or 3-aminoacetophenone (**2** or **3**), subjecting the mixture to refluxing (80-85 °C) with constant stirring for 9 hours. The resulting products 4-[(7-chloroquinolin-4-yl)amino]acetophenone (**4**) and 3-[(7-chloroquinolin-4-yl)amino]acetophenone (**5**) were filtered, washed with ethanol and diethyl ether. These ligands were recrystallized from a mixture of ethanol-methanol (2:1). The reaction route is shown in Scheme 1.

### Synthesis of metal complexes

A solution of copper (II) chloride (0.02 M and 25 mL MeOH) was added to a methanol solution of free ligand (0.02 M and 25 mL MeOH). The reaction mixture was refluxed on a sand bath for 17 hours at 60 °C with constant agitation and inert atmosphere (Ar). In both cases, solid-colored complexes were obtained and collected by filtration, washed on paper with cold methanol and diethyl ether, dried under vacuum in an oven at 40 °C. The purity of the complexes was checked by TLC.

### Antimalarial activity 

#### Inhibitory activity against in vitro growth of Plasmodium falciparum (W2)

The antimalarial activity was evaluated using the protocol described by Chipeleme et al. (2007[5]). The controls used were chloroquine, artemisinin and CuCl_2_. Parasitemia was determined by fluorescence plots using flow cytometry (CellQuest software, Becton Dickinson). Percentile plots of parasitemia versus compound concentrations were used to obtained IC_50_. The data were fit to a nonlinear regression analysis using GraphPad Prism 3.02 software (Reinders et al., 1995[36]). For statistical analyses, Student's t-tests were performed, and significance was set at *p* < 0.05. 

#### Inhibition of hemozoin formation (β-hematin)

The assay of inhibition of *β-*hematin formation (synthetic hemozoin) was developed according to the protocol described by Baelmans et al. (2000[2]). Only the free ligands were considered in the inhibitory test where chloroquine was the control. Results are expressed as percentage of inhibition of β-hematin formation (% IβHF). The significance was set at *p* > 0.05 compared to chloroquine. 

#### In vitro inhibitory activity against recombinant falcipain-2

The IC_50_ values against the recombinant enzyme, falcipain-2, were determined from the plots of activity percentage over the compound concentrations; the data was fit to a nonlinear regression analysis using GraphPad Prism 3.02 software. The controls used were the cysteine protease inhibitor E-64 [N-(trans-epoxysuccinyl)-1-leucine-4-guanidinobutylamide] and CuCl_2_ (Rosenthal et al., 1996[37]; Shenai et al., 2000[38]). For statistical analyses, Student's t-tests were performed, and significance was set at p < 0.05.

**
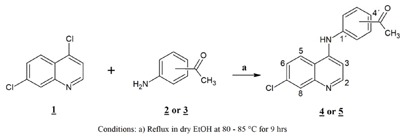
**

**Scheme 1:** Synthesis of [(7-chloroquinolin-4-yl)amino]acetophenones (**4**, **5**)

### Computational section

#### Computational details

The molecular structure of compounds **4a** and **5a** have been elucidated theoretically with Gaussian 09 program using the DFT (B3LYP) level of theory at 6-31G (d,p) basis set (Frisch et al., 2009[14]). The harmonic vibrational frequencies were calculated at the same levels of theory for the optimized structure and the vibrational band assignments were made using the Gauss-View molecular visualization program (Dennington et al., 2007[9]). The nonexistence of imaginary wavenumbers of the theoretically calculated vibrational spectra affirms that these deduced structures correspond to minimum energy. However, the wavenumber values obtained at these levels contain well-known systematic errors (Foresman and Frisch, 1996[13]). Therefore, the calculated vibrational wavenumbers were scaled with a scale factor of 0.9614 for B3LYP method (Young, 2001[51]).

#### Molecular docking procedure

The ligands in protonated form (**4** and **5**) were built using the molecule builder of the software package ArgusLab 4.0.1. Geometry optimizations and energy calculations of the ligands were performed using the PM3 semi-empirical quantum-mechanical method. The binding site was the three-dimensional structure of the hemozoin dimer (*β-hematin*), and it was obtained from the Cambridge Crystallographic Database REFCODE: XETXUP (Pagola et al., 2000[27]). Hemozoin dimer was solvated in order to simulate experimental conditions of the *in vitro* evaluations, considering only those water molecules that establish H-bonds with the hemozoin dimer. The docking program implements an efficient grid-based docking algorithm which approximates an exhaustive search within the free volume of the binding site solvated, around which was built a bounding box (25 x 25 x 25 Å). It was used ArgusDock engine, and the conformational space was explored by the geometry optimization of the flexible ligand under a condition of high precision docking. The final positions of the compounds were ranked by lowest interaction energy values (docking score in kcal mol^-1^). Hydrogen bond interactions, as well as favorable π-π interactions between the ligands and hemozoin dimer solvated, were explored.

## Results and Discussion

### Characterization of ligands (L)

**4-[(7-chloroquinolin-4-yl)amino]acetophenone** (**4**). Pale yellow amorphous compound, yield: 94.66 %, m.p. 288-290 °C, R_f_ (Hexane:ethyl acetate = 3:7): 0.76. UV-vis (DMSO) [λ_max_ (nm), ε (cm^-1^ mol^-1^ L), assignment]: 261, 2100, aromatic π→π*; 362, 2792, π→π* and/or n→π* (conjugated carbonyl). IR (KBr) cm^−1^: 3440, 1678, 1623, 1588, 1098. ^1^H NMR (DMSO-d_6_): δ 2.62 (s, 3H, CH_3_), 7.04 (d, 1H, H_3_, J_2,3_: 6.7 Hz), 7.66 (d, 2H, H_3´,5´_, J_2´,3´_ = J_5´,6´_: 8.4 Hz), 7.89 (dd, 1H, H_6_, J_5,6_: 9.2 Hz, J_6,8_: 1.7 Hz), 8.12 (d, 2H, H_2´,6´_, J_2´,3´_ = J_5´,6´_: 8.4 Hz), 8.22 (d, 1H, H_8_, J_6,8_: 1.7 Hz), 8.62 (d, 1H, H_2_, J_2,3_: 6.9 Hz), 8.95 (d, 1H, H_5_, J_5,6_: 9.2 Hz), 11.39 (broad s, 1H, NH). ^13^C NMR: δ 27.21 (CH_3_), 102.2, 117.42, 120.73, 124.50, 126.99, 127.84, 130.44, 135.22, 138.43, 140.86, 142.67, 145.06, 154.14 (Aromatic-C), 197.30 (C=O). HRMS *m/z* (ESI): 297.00 [M+H]^+^, exact mass calculated for C_17_H_14_ClN_2_O [M+H]^+^: 297.00, found: 297.00; ESI-CID-MS^2^: 297.00 (21) [M+H]^+^, 253.99 (100), 218.14 (16), 163.10 (3), 137.97 (≈ 1), 111.07 (≈ 1), 91.15 (≈ 1), 77.06 (≈ 1), 43.17 (≈ 1).

**3-[(7-chloroquinolin-4-yl)amino]acetophenone** (**5**). Pale yellow crystalline compound, yield: 97.92 %, m.p. 294 °C, R_f_ (Hexane:ethyl acetate = 3:7): 0.73. UV-vis (DMSO) [λ_max_ (nm), ε (cm^-1^ mol^-1^ L), assignment]: 259, 2351, aromatic π→π*; 347, 2335, π→π* and/or n→π* (conjugated carbonyl). IR (KBr) cm^−1^: 3440, 1685, 1611, 1581, 1100. ^1^H NMR (DMSO-d_6_): δ 2.62 (s, 3H, CH_3_), 6.87 (d, 1H, H_3_, J_2,3_: 6.9 Hz), 7.75 (m, 2H, H_4´,6´_), 7.90 (dd, 1H, H_6_, J_5,6_: 9.2 Hz, J_6,8_: 1.97 Hz), 8.01 (t, 1H, H_5´_, J: 7.4 Hz), 8.04 (s, 1H, H_2´_), 8.21 (d, 1H, H_8_, J_6,8_: 2.2 Hz), 8.55 (d, 1H, H_2_, J_2,3_: 6.9 Hz), 8.95 (d, 1H, H_5_, J_5,6_: 9.2 Hz), 11.39 (broad s, 1H, NH). ^13^C NMR: δ 27.3 (CH_3_), 102.8, 119.0, 121.9, 124.3, 125.1, 125.8, 127.2, 127.9, 130.4, 134.8, 138.7, 141.4, 148.4, 149.8, 152.2 (Aromatic-C), 198.2 (C=O). HRMS *m/z* (ESI): 296.95 [M+H]^+^, exact mass calculated for C_17_H_14_ClN_2_O [M+H]^+^: 297.00, found: 296.95; ESI-CID-MS^2^: 297.00 (28) [M+H]^+^, 253.01 (100), 218.03 (33), 163.01 (5), 137.97 (≈ 1), 110.99 (≈ 1), 91.09 (≈ 2), 77.09 (≈ 1), 43.13 (≈ 1).

Spectral data confirmed the proposed molecular and structural formula for the ligands of kind acetophenone (Scheme 1). Supplementary Figures 1, 5, 7, 11 illustrate the NMR spectra. The spectra of compounds registered signals due to the presence of ethanol used in the purification process.

#### Mechanisms of mass spectral fragmentation

The study of compounds **4** and **5** through electrospray ionization (ESI), followed by tandem mass experiments (MS^2^) with collision-induced dissociation (CID), at a voltage of 40-45 eV and in mode positive; generated interesting mass spectrometry results.

The elucidation of the precursor molecular ion fragmentation pathways [**4** + H]^+^ and [**5** + H]^+^ allowed us to understand the dynamics of the protonation of the 4-amino-7-chloroquinoline nucleus in the presence of the acetyl group, the protonation of the thermodynamically favored position does not always trigger the processes of fragmentation (Ya-Ping, 2012[49]). For this reason, it is convenient to differentiate between the terms protonation site(s) and dissociative protonation site(s) (Ya-Ping, 2006[50]).

**Figure 1:**
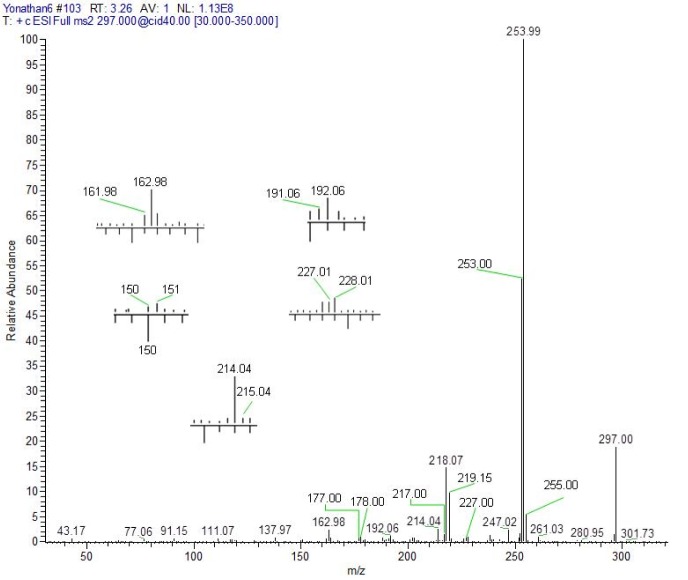
CID Product ion spectrum of protonated 4 by MS-MS

**Figure 2:**
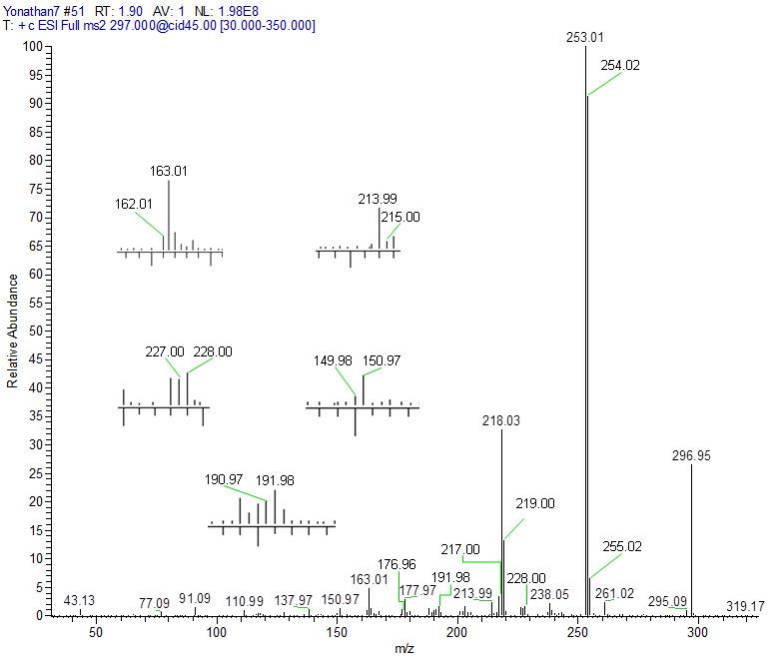
CID Product ion spectrum of protonated 5 by MS-MS

**Figure 3:**
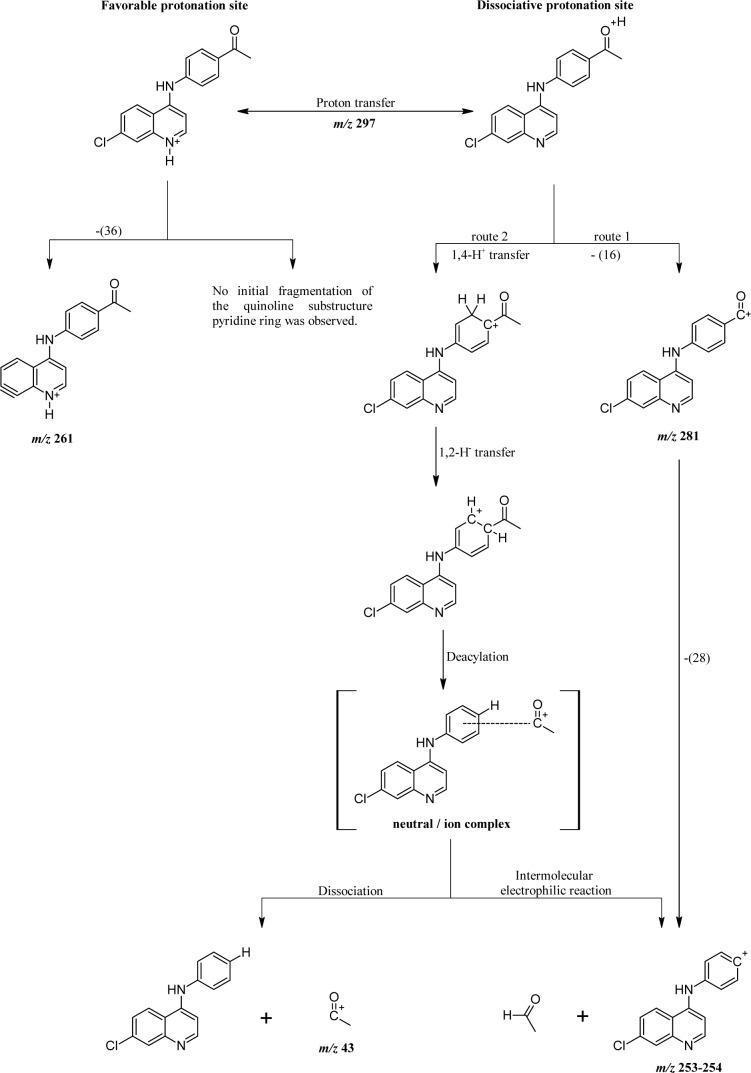
Proposed fragmentation mechanisms for precursor molecular ion [4 + H]^+^ (shown) or [5 + H]^+^. Note: numbers in parentheses represent mass loss due to neutral molecules.

The fragments observed in the MS^2^ spectra of compounds **4** (Figure 1(Fig. 1)) and **5** (Figure 2(Fig. 2)) were similar, recording a signal at *m/z* 297 corresponding to the precursor molecular ion [C_17_H_13_ClN_2_O + H]^+^. This ion was probably protonated in the quinolinic nitrogen, the most favorable thermodynamic position according to the proton affinity values (Hunter and Lias, 1998[19]). Under induced collision, the molecular ion protonated in nitrogen at *m/z* 297, only released one molecule of HCl (36 Da) but did not generate any fragment indicating the decomposition of the pyridine substructure of the quinoline ring (Figure 3(Fig. 3)).

The main fragmentation reaction begun with the deacylation by heterolytic cleavage of the alpha (α) bonds to the carbonyl. This pathway was possible if one considers the migration of a proton from the initial protonation site (quinolinic-N) to the site of dissociative protonation. So, proton transfer is proposed before fragmentation occurs (Thielking et al., 1992[43]; Pengyuan et al., 2010[32]; Demarque et al., 2016[8]).

After proton transfer (Figure 3(Fig. 3)), the acetyl group underwent two routes of fragmentation reactions, which differed in the number of breaks of α-bonds to the carbonyl. Route 1 was given by cleavage of the methyl-carbonyl α-bond, releasing methane (16 Da) and producing the ion at *m/z* 281, in turn, this fragment generated the ion at *m/z* 253-254 by loss of a mass of 28 Da (carbon monoxide). Route 2 implies a process of fragmentation that is explained through the formation of a neutral/ion complex, but where apparently two mechanisms competed, one of dissociation (Thielking et al., 1992[43]; Filges and Grützmacher, 1987[12]) and another of hydride abstraction by an intermolecular electrophilic reaction (Thielking et al., 1992[43]; Wenthold and Liu, 2001[46]). The first mechanism generated the observed acyl ion at *m/z* 43, while the second mechanism provided the main product ion at *m/z* 253-254.

**Figure 4:**
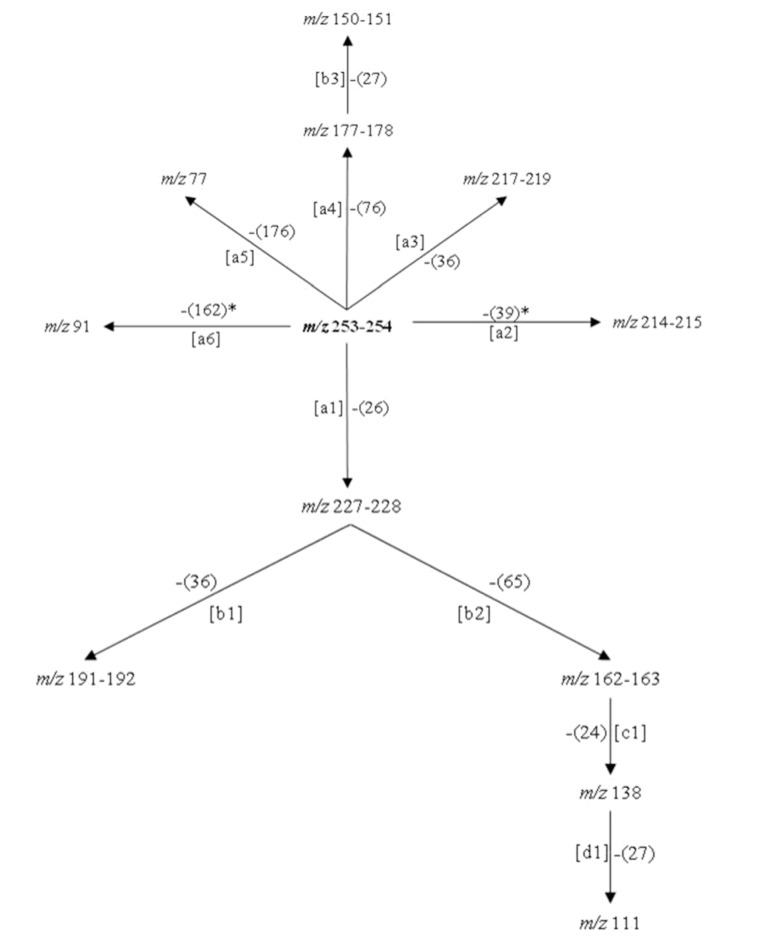
Proposed fragmentation scheme from the product ion at *m/z* 253-254. Note: [ ]: fragmentation path, *: free radical path, ( ): neutral residual fragment.

**Figure 5:**
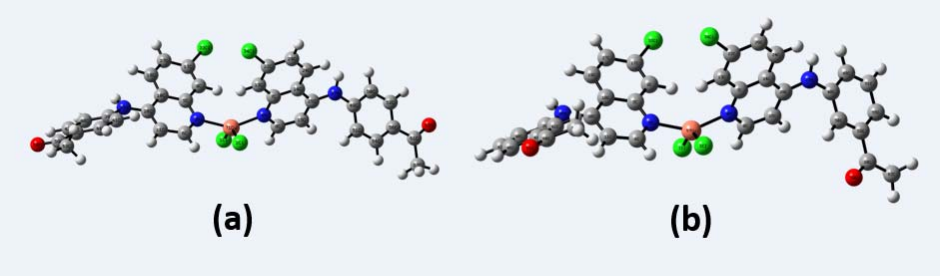
Optimized structures of the compounds 4a (a) and 5a (b) on B3LYP/6-31G(d, p) basis set

As can be seen in the spectra of compounds **4** (Figure 1(Fig. 1)) and **5** (Figure 2(Fig. 2)), the signals at *m/z* 253, 254 and 255; suggest different isotopic distributions due to the presence of heteroatoms in the structure. Figure 4(Fig. 4) shows the proposed fragmentation from the ion at *m/z* 253-254 (Supplementary Figures 13-16), with the probable structures of descendant fragments (Table 1(Tab. 1)).

### Characterization of the Cu(II) complexes

**{4-[(7-chloroquinolin-4-yl)amino]acetophenone}copper(II) chloride (4a). **Dark green solid compound, m.p. 228-230 °C, IR (KBr) cm^−1^: 3440, 1676.8, 1619.2, 1584, 1094. EPR (solid): g_iso_ = 2.1658. ^1^H NMR (DMSO-d_6_): δ 2.59 (s, 3H, CH_3_), 6.53 (d, 1H, H_3_, J_2,3_: 8.2 Hz), 7.61 (d, 2H, H_3´,5´_, J_2´,3´_ = J_5´,6´_: 7.8 Hz), 7.81 (dd, 1H, H_6_, J_5,6_: 8.7 Hz), 8.08 (d, 2H, H_2´,6´_, J_2´,3´_ = J_5´,6´_: 7.8 Hz), 9.34 (broad s, 1H, H_5_), 10.92 (broad s, 1H, NH). ^13^C NMR: δ 27.23 (CH_3_), 112.85, 124.11, 127.30, 128.03, 130.53, 134.99, 138.08, 142.89 (Aromatic-C), 197.25 (C=O). ESI-CID-MS^2^: [M(L)_2_]^+^ = [^65^Cu(C_17_H_13_ClN_2_O)_2_]^+^ = 657.18 (38). Selected fragments of [M(L)_2_]^+^ = 378.93 (50), 360.87 (100), 280.99 (10), 260.87 (7).

**{3-[(7-chloroquinolin-4-yl)amino]acetophenone}copper(II) chloride** (**5a**). Light green crystalline compound, m.p. 224 °C, IR (KBr) cm^−1^: 3440, 1680, 1613, 1578, 1100. EPR (solid): g_x_ = 2.3436, g_y_ = 2.1622, g_z_ = 2.1055. ^1^H NMR (DMSO-d_6_): δ 2.63 (s, 3H, CH_3_), 6.92 (broad s, 1H, H_3_), 7.74 (m, 2H, H_4´,6´_), 7.89 (d, 1H, H_6_, J_5,6_: 8.1 Hz), 8.00 (m, 2H, H_2´,5´_), 8.16 (broad s, 1H, H_8_), 8.85 (d, 1H, H_5_, J_5,6_: 9.1 Hz), 11.1 (broad s, 1H, NH). ^13^C NMR: δ 27.4 (CH_3_), 100.0, 125.2, 126.6, 127.8, 128.1, 130.3, 131.1, 138.2, 139.1 (Aromatic-C), 197.8 (C=O). ESI-CID-MS^2^: [M(L)_2_]^+^ = [^65^Cu(C_17_H_13_ClN_2_O)_2_]^+^ = 657.44 (19). Selected fragments of [M(L)_2_]^+^ = 378.94 (9), 360.92 (100).

#### Molecular structures

The computational calculations of the compounds **4a** and **5a**, including geometry optimization, single point energies and vibrational wavenumbers evaluations; were performed by using Gaussian 09 program at B3LYP/6-31G (d,p) level. The B3LYP is a solid and efficient exchange correlation function for calculating energies and geometries; it has been proven to be able to yield reliable results for small molecules. 

The difference in experimental and calculated geometrical parameters came from the environment of the compound. It is clear that the experimental results belong to solid phase, and the theoretical calculations have been performed in the gas phase and the intermolecular interactions are not taken into account. The molecular structure of the compounds **4a** and **5a** with atom numbering scheme adopted in the computations is shown in Figures 5a and 5b(Fig. 5), respectively.

#### FTIR analysis

The FTIR spectra of both compounds were recorded in the range of 4000-400 cm^-1^ and calculated for the optimized structures at the DFT/B3LYP method with the 6-31G (d,p) basis set. Both compounds have 71 atoms with 207 normal modes of vibrations.

In the experimental FTIR spectra of both compounds was possible to observe the absorption band 3440 cm^-1^ due to NH stretching, whereas this vibration corresponded to 3635.40 cm^-1^ and 3637.59 cm^-1^ in the calculated FTIR spectra of the compounds **4a** and **5a**, respectively.

The absorption band corresponding to C=O stretching vibration of the ketone group was observed at 1676.8 cm^-1^ in the experimental IR spectrum and 1616.79 cm^-1^ in the calculated IR spectrum for compound **4a**. In compound **5a**, this stretching was also present at 1680 cm^-1^ and 1661.13 cm^-1^ in the experimental and theoretical spectra, respectively.

The absorption band associated with C=N bond stretching, for compound **4a**, appears at 1584 cm^-1^ in the experimental IR spectrum and 1551.63 cm^-1^ in the theoretical spectrum; whereas for compound **5a**, these are observed at 1577.6 cm^-1^ and 1562.81 cm^-1^, respectively. The C-N stretching band appeared at 1356 cm^-1^ and 1351.15 cm^-1^ in the experimental and theoretical spectra of compound **4a**, and in the compound **5a,** 1360 cm^-1^ and 1359.94 cm^-1^. 

Finally, the absorption band corresponding to C-Cl stretching, for compound **4a**, appeared at 1094 cm^-1^ in the experimental spectrum and 1091.88 cm^-1^ in the theoretical spectrum. In compound **5a**, the same signal was observed at 1100 cm^-1^ in the experimental spectrum and 1090.32 cm^-1^ in the theoretical spectrum.

#### Mechanisms of mass spectral fragmentation of the Cu(II) complexes

The experimental conditions during the electrospray ionization process (ESI) favored the partial or complete dissociation of the uncoordinated counterions, but also of those weakly coordinated to the metal, registering in the spectrum only the cationic fragment (Wikstrom et al., 2010[48]; Underwood et al., 2013[44]).

**Figure 6:**
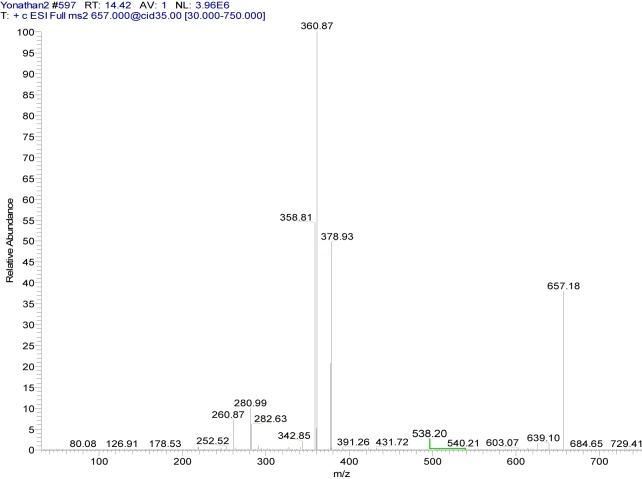
Positive ion mode ESI-CID-MS^2^ spectrum of the ion [^65^Cu(C_17_H_13_ClN_2_O)_2_]^+^ at *m/z* 657.18 (cationic unit of 4a complex)

**Figure 7:**
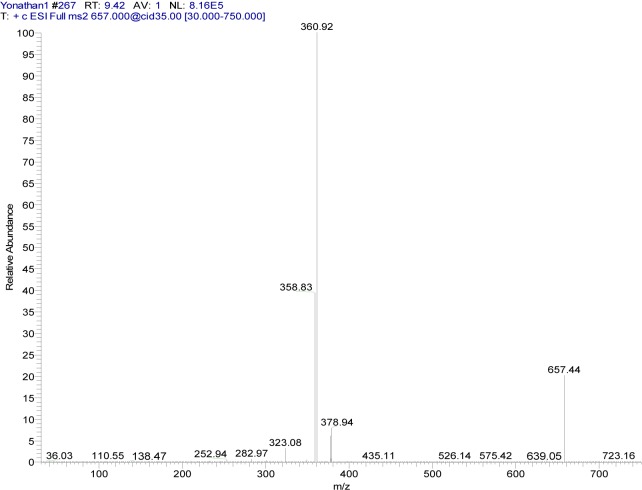
Positive ion mode ESI-CID-MS^2^ spectrum of the ion [^65^Cu(C_17_H_13_ClN_2_O)_2_]^+^ at *m/z* 657.44 (cationic unit of 5a complex)

The MS^2^ spectra of compounds **4a** (Figure 6(Fig. 6)) and **5a** (Figure 7(Fig. 7)) showed slight differences that reveal changes in fragmentation patterns, which is inferred to be a consequence of isomerism between ligands **4** and **5**. However, both indicated the cationic fragment of the coordination complexes was the [Cu(L)_2_]^+^ type, registering a signal at *m/z* 657; they corresponded to the precursor molecular ion [Cu(C_17_H_13_ClN_2_O)_2_]^+^.

The center of positive charge was on the metal, but contrary to the expected according to the electronic paramagnetic resonance data (Figures 10 and 11), where the paramagnetic character of the complex was shown, and therefore, the oxidation state Cu(II) was confirmed. In gaseous phase, the metal is in the Cu(I) form; this is indicated by the determination of the state of charge of the signals in the spectra where different charge distributions are not observed. These electronic differences suggest that copper underwent a reduction during ionization by electrospray, which is reasonable considering the experimental conditions.

The solutions of these complexes were prepared in mixtures of dimethylsulfoxide:methanol (v/v 1:2) and dispersed by applying a very high voltage (2-6 kV), this strong electric field exceeded the ionization energy of the molecules of DMSO of 9.1 eV (Kimura et al., 1981[20]) and MeOH of 10.85 eV (Tao et al., 1992[42]). Thus, an electron could be transferred from the neutral solvent molecules to the ion of the doubly charged metal Cu(II). This is a favorable-energetically reduction process because this metal has high second ionization energy (20.3 eV) (Haynes, 2012[17]; Shi et al., 2006[39]; Park et al., 2007[28]), which is higher than all the transition metals.

On the other hand, copper has two isotopes (^63^Cu and ^65^Cu) with a natural abundance ratio of 69.09:30.91 (Haynes, 2012[17]). In this sense, Figures 6(Fig. 6) and 7(Fig. 7) show the CID-MS^2^ spectra specifically for the precursor molecular ion [^65^Cu(C_17_H_13_ClN_2_O)_2_]^+^. However, as noted below, additional signals were indicating the presence of the ^63^Cu isotope.

**Figure 8:**
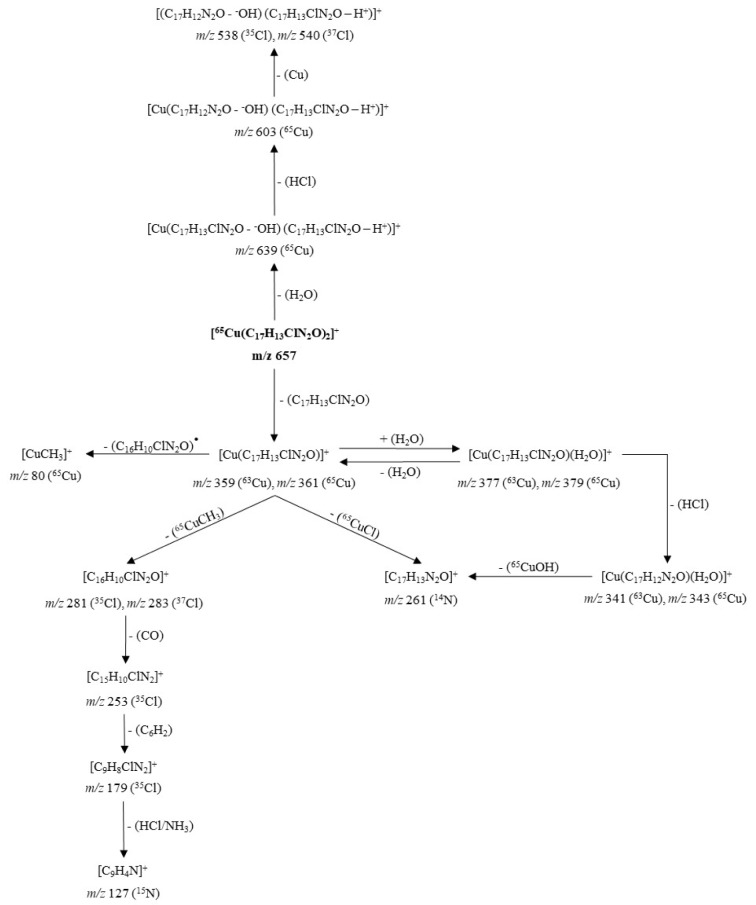
Proposed fragmentation for the ion [^65^Cu(C_17_H_13_ClN_2_O)_2_]^+^ at *m/z* 657 (cationic unit of 4a complex)

The pathways of fragmentation observed for the cationic unit of the complex **4a** and its product ions are shown in Figure 8(Fig. 8). The dissociation induced at 35 eV of [^65^Cu(C_17_H_13_ClN_2_O)_2_]^+^ (*m/z* = 657) resulted in two channels. The minority channel (Supplementary Figure 17) begun with the tautomerization of each ligand up to its enol form, with subsequent inter-ligand proton transfer accompanied by dissociation, promoting the loss of an H_2_O molecule to generate the ion at *m/z* 639. This ion lost HCl to give the fragment at *m/z* 603, which released neutral copper by charge reduction through the transfer of an electron from the ligand to the Cu(I) metal ion, producing the fragment at *m/z* 538 (^35^Cl ) - 540 (^37^Cl).

This mechanism of dissociation by tautomerization and subsequent loss of H_2_O, suggests that the carbonyl groups did not act as coordination sites to the metal, a property that is in agreement with that found by ^1^H NMR and ^13^C NMR.

The dominant channel (Supplementary Figure 18) involved the loss of a neutral ligand molecule to generate the ion at *m/z* 359 (^63^Cu) - 361 (^65^Cu). This ligand released without prior inter-ligand proton transfer or electron transfer from the ligand to the metal, suggesting that the driving force of the dissociation was different from the coulombic repulsions. Consequently, it is proposed that steric repulsions promoted dissociation; these interactions were more intense if it is considered that both ligands were coordinated to the metal by means of the quinolinic nitrogen atoms.

The fragment at *m/z* 359-361 underwent three dissociation pathways and a reversible re-coordination/de-coordination of a water molecule (Supplementary Figures 18 and 19). A dissociative path occurred by homolytic cleavage of the methyl-carbonyl α-bond of the ligand, releasing the neutral ligand and a methyl-copper at *m/z* 80. Another dissociation proceeded by heterolytic cleavage of the chloro-quinoline bond, generating the chloride anion (Cl^-^) which was spontaneously driven up to the metal ion Cu(I) finally losing CuCl and producing the ion at *m/z* 261. This product was also generated from the fragment at *m/z* 341 (^63^Cu) - 343 (^65^Cu) by liberation of CuOH. 

The last dissociation pathway, characterized by its extensive fragmentation, was triggered by the heterolytic cleavage of the methyl-carbonyl α-bond of the ligand, generating the methyl carbanion (^-^CH_3_) that reacts with the Cu(I) ion, releasing the neutral CuCH_3 _and yielding the cation at *m/z* 281 (^35^Cl) - 283 (^37^Cl). This fragment formed the ion at *m/z* 253 by the loss of a molecule of CO, which in turn, lost the neutral 1,2,3,4-tetradehydrobenzene, and formed the ion at *m/z* 179. Finally, this ion underwent a double simultaneous dissociation releasing HCl and NH_3_ to produce the cation at *m/z* 127, corresponding to 7,8-didehydroquinolin-4-ilium.

**Figure 9:**
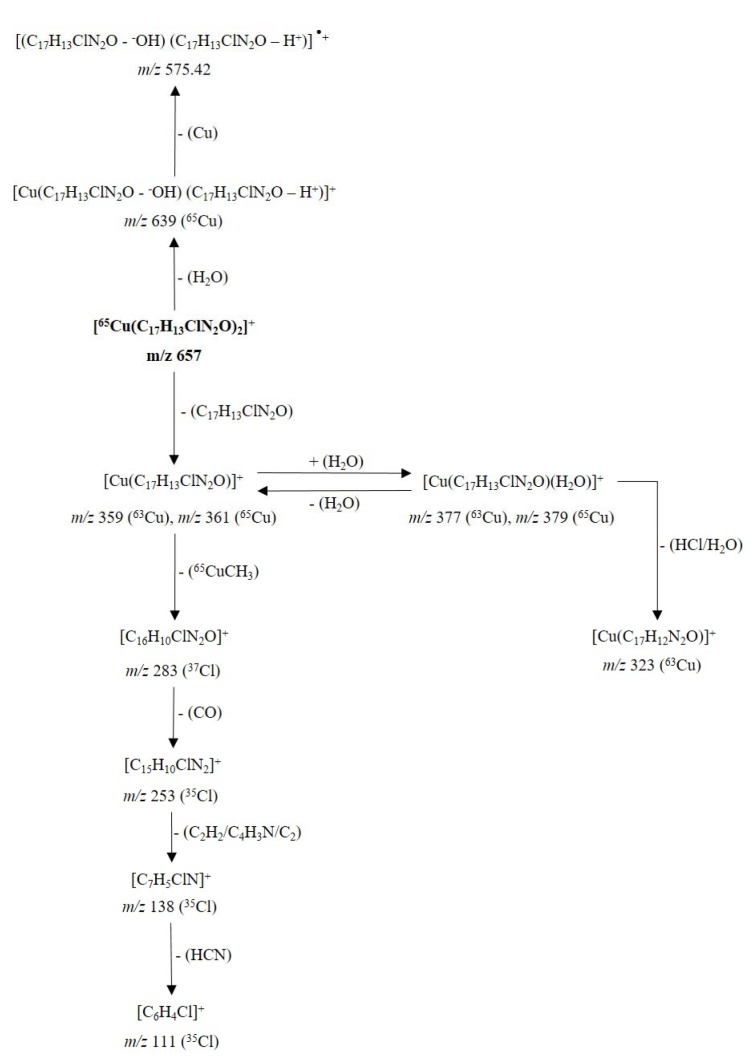
Proposed fragmentation for the ion [^65^Cu(C_17_H_13_ClN_2_O)_2_]^+^ at *m/z* 657 (cationic unit of 5a complex)

The fragmentation mechanism of the cationic unit of the coordination complex **5a** (Figure 9(Fig. 9)) it is not discussed in detail, due to the presence of some dissociations, equivalent to those shown for the cationic fragment of the complex **4a**.

#### NMR spectral studies of the Cu(II) complexes

The ^1^H NMR spectra of the complexes** 4a** (Supplementary Figure 2) and **5a** (Supplementary Figure 8) indicate that the quinoline ring protons were significantly affected by the effect of the paramagnetic metal ion. For the complex **4a**, the signals assignable to H2 and H8 were absent, while the complex **5a** did not show H2 signal, but if the one assigned to H8 with a pronounced widening and diminution of its intensity (Table 2(Tab. 2)). The protons of the phenyl ring and the methyl do not undergo significant changes; for these reasons, the quinolinic nitrogen atom must be involved in the coordination. The above statement is confirmed by the study of ^13^C NMR spectra (Supplementary Figures 6 and 12), where both compounds recorded spectra with disappearances of signals due to resonances of carbon atoms of the quinoline ring (Table 2(Tab. 2)).

#### Electronic paramagnetic spectra of the Cu(II) complexes

**Figure 10:**
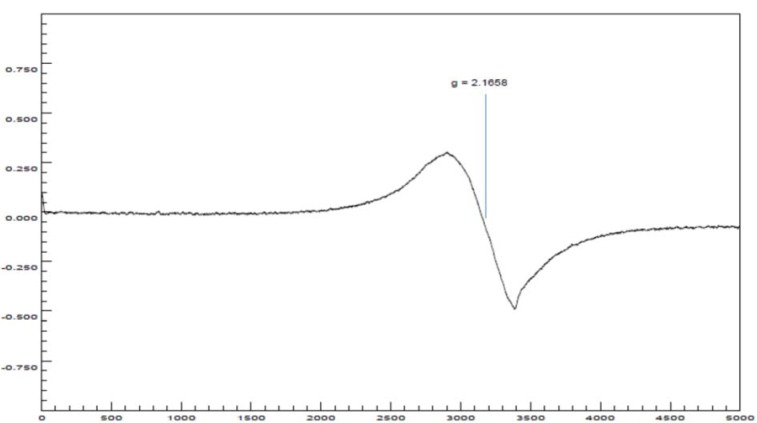
X-band EPR spectrum of the polycrystalline complex 4a at 298 K

The EPR spectrum at room temperature for coordination complex **4a** (Figure 10(Fig. 10)) showed an isotropic signal without any hyperfine splitting, due to dipolar interactions between magnetic moments (electronic and nuclear) (Zhidomirov et al., 1969[52]) and increased spin-red relaxation (Hoffmann et al., 1996[18]), with a value of *g* o *g*_iso_ = 2.1658. The value of *g*_iso_ tensor obtained in this study, indicated an increase in the covalence of a bond between the metal ion Cu(II) and the ligand molecules, when compared with the value of *g* for the free electron (*g_e_-* = 2.0023) (Angelusiu et al., 2008[1])

**Figure 11:**
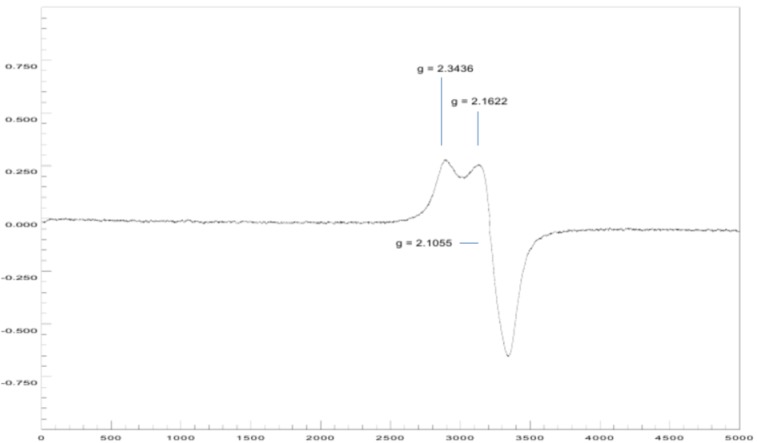
X-band EPR spectrum of the polycrystalline complex 5a at 298 K

The EPR spectrum at room temperature for coordination complex **5a** (Figure 11(Fig. 11)) shows a rhombic symmetry, with three values of the tensor g of, *g*_x_ = 2.3436, *g*_y_ = 2.1622 and *g*_z_ = 2.1055, where the lowest value of *g* is higher than 2.04 (*g*_z_ > 2.04). The geometric parameter G for this complex shows a value of less than 4; this suggests significant exchange interactions between centers metal in the solid state, which is probably a consequence of the closeness between independent molecules of the complex (Hathaway and Billing, 1970[16]).

The spectral studies revealed that Cu(II) ion in both complexes was in a tetragonal field, with the following probable stereochemistries: for complex **4a**, square planar (Sreekanth and Prathapachandra Kurup, 2003[41]) or distorted tetrahedron (Murphy and Hathaway, 2003[25]) with the unpaired electron in the orbitals dx^2^-y^2^ and dxy, respectively; for complex **5a**, rhombic square coplanar with the unpaired electron in the dx^2^-y^2^ orbital. However, steric and electronic factors associated with the presence of four bulky ligands and sigma (σ) donors, make strictly planar or square coplanar stereochemistries almost impossible (Hathaway and Billing, 1970[16]).

#### Relationship between spectroscopy, spectrometry and structure of Cu(II) complexes

The structure-spectroscopy relationship for the compounds **4a** and **5a** indicates that the atom involved in the coordination corresponded to the endocyclic nitrogen of the quinoline ring, according to the infrared region; the assignment was linked to the displacement of the aromatic C=C vibrations [1622 cm^-1^ (**4**) → 1619 cm^-1^ (**4a**); 1613 cm^-1^ (**5**) → 1608 cm^-1^ (**5a**)] which suggested that the Cu(II) ion must be associated with a ring member atom rather than the carbonyl group. The spectra of ^1^H NMR and ^13^C NMR showed widening and/or decrease of the signal intensity of the protons and carbon atoms of the quinoline ring, while the signals of the phenyl ring and the acetyl group remained unchanged.

The ESI-CID-MS^2^ experiments reveal an ion of formula [^65^Cu(C_17_H_13_ClN_2_O)_2_]^+^, whose fragmentation pattern was following the previous assignments, in addition to suggesting a coordination number of two and an oxidation state 1+ for the metal. These last two observations are opposed to the solid phase EPR studies, which highlights the paramagnetic nature of the compound and a tetragonal environment around the Cu(II) ion, with square planar geometries or distorted tetrahedron (**4a**) and square rhombic coplanar (**5a**). Therefore, these remarkable differences point to a complete dissociation of counterions and a reduction of Cu(II) → Cu(I) during the ESI process; the identity of counterions can be assigned to chloride anions since the salt used was in the form of CuCl_2_.

The spectroscopic correlations show that these coordination complexes were of the mononuclear-tetracoordinate type, with a metal-ligand stoichiometry of 1:2, where both ligand molecules behaved as monodentate in neutral form. The charge of Cu(II) ion was neutralized by two chloride anions which were semi-coordinated (Hathaway and Billing, 1970[16]), in such a way that they affected the coordination sphere of metallic ion in the solid state, but dissociated in solution. In this sense, the general formulae for these complexes can be expressed in two ways, according to the phase in which they are: [Cu(L)_2_Cl_2_] for solid phase and [Cu(L)_2_][2Cl] for the liquid phase.

### Computational analysis of the proposed molecular structures of Cu(II) complexes

#### Analysis of frontier molecular orbitals (FMOs)

**Figure 12:**
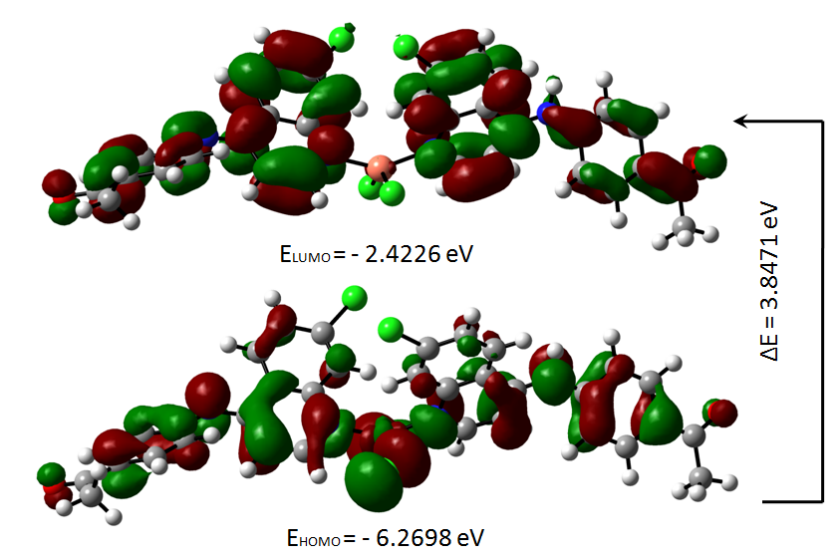
Molecular orbitals and energies for the HOMO and LUMO of compound 4a

**Figure 13:**
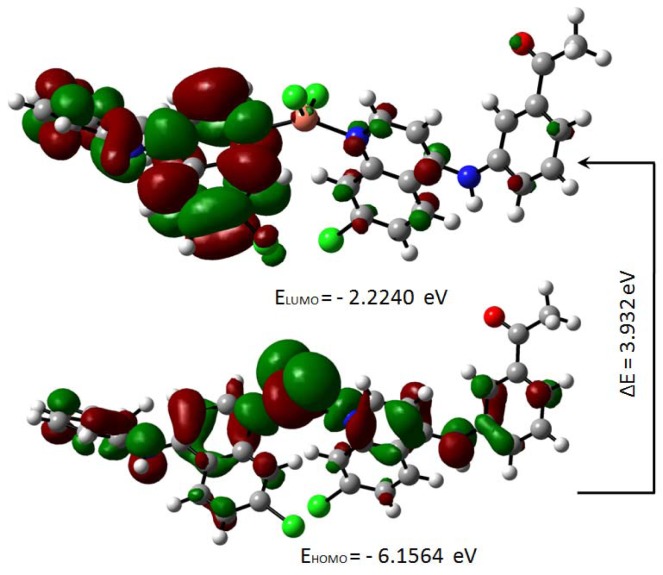
Molecular orbitals and energies for the HOMO and LUMO of compound 5a

The highest occupied molecular orbital (HOMO) and the lowest unoccupied molecular orbital (LUMO) are useful descriptors in studying chemical stability of molecules (Pearson, 1989[31]). The energy of HOMO and LUMO characterize the ability of electron donating and accepting, respectively. Both HOMO and LUMO were calculated at the B3LYP/6-31G(d,p) level in order to evaluate the energetic behavior of both title molecule. The energies and the pictorial illustration of HOMO and LUMO frontier molecular orbitals are shown in Figures 12(Fig. 12) and 13(Fig. 13); the positive and negative phase is represented in red and green color, respectively. 

The HOMO-LUMO plot clearly elucidates the eventual charge transfer taking place in the crystal structure. In the HOMO plot of compound **4a**, the charge was localized on C13, N37, N4, C56, O62, C9, N3, N35, C46, O60, Cu71, Cl1 and Cl2; and localized on the bonds C10-C20-C11, C39-C40-C42, C14-C25-C15, C50-C49-C51, C19-C27 and C29-C24. The LUMO was spread over the entire molecule except for the hydrogen atoms and principally localized on the bonds C59-C46, C44-C41, C42-C40, C39-N35, C11-C6, C27-C17, C8-C15, C22-C29, N37-C49, C51-C54, C50-C52, C56-C61 and the atoms N3, C20, O60, C19, C9, N4, C25, C13, C24 and O62. In the HOMO plot of compound **5a**, the charge was located over the bonds C40-C39, C19-C27, C25-C15, C48-C49, C20-C11-C10 and the atoms C46, C41, N35, C9, N3, C14, N4, C13, N37, C50, C55, Cu57, Cl2 and Cl1; whereas the LUMO was located over the bonds C11-C6, C27-C17, C39-N35 and C60-C63 and the atoms C20, C22, C8, C9, C40, C49, C55, C25, C13, C24, N3, O59, O61, N4.

The HOMO-LUMO gap, predicting the energy difference between HOMO and LUMO is an essential stability index (Reed et al., 1988[35]). A large HOMO-LUMO gap of a molecule implied greater stability and lesser reactivity in chemical reactions. On calculating the HOMO-LUMO energy gap for compounds **4a** and **5a**, the values were 3.847 eV and 3.932 eV, respectively. The conjugated molecules are characterized by a highest occupied molecular orbital-lowest unoccupied molecular orbital (HOMO-LUMO) separation, which is the result of a significant degree of intermolecular charge transfer (ICT) from the end-capping electron-donor to the efficient electron acceptor group through *p* conjugated path. These values explained the eventual charge-transfer interaction within the molecule, which influenced the biological activity of the compound. The HOMO-LUMO energies and related parameters governing chemical reactivity and molecular stability are shown in Table 3(Tab. 3).

For understanding the various aspects of the characteristics of the bioactive molecules, several new chemical reactivity descriptors have been proposed. Conceptual DFT based descriptors have helped in many ways to understand the structure of molecules and their reactivity by calculating the chemical potential, global hardness and electrophilicity. The HOMO and LUMO orbital energies, ionization energy (I), electron affinity (A), global hardness (η), chemical potential (μ) and electrophilic index (ω) (Parr et al., 1999[29]) of the compounds **4a** and **5a** can be calculated and is shown in Table 3(Tab. 3).

#### Thermodynamic parameters

Thermodynamic parameters, namely, SCF energy, entropy (S), enthalpy (H), Gibbs free energy (G), zero-point energy (E0) are determined using DFT method employing B3LYP functional with 6-31G (d,p) basis set. All the thermodynamic calculations were done in the gas phase (Table 3(Tab. 3)). These calculated thermodynamic data are useful in predicting the reactivity of chemical reactions and judging the feasibility of different reaction pathways.

#### Determination of molecular electrostatic potential (MEP)

Recently, the MEPs have been used for interpreting and predicting relative reactivities sites for electrophilic and nucleophilic attack, investigation of biological recognition, hydrogen bonding interactions, studies of zeolite, molecular cluster and crystal behavior, and the correlation and prediction of a wide range of macroscopic properties (Pirnau et al., 2008[33]; Murray and Sen, 1996[26]).

In order to grasp the molecular interactions, molecular electrostatic potentials (MEPs) of compounds **4a** and **5a** have been determined. It gives an idea about the charge distribution and the relationship between dipole moments, electronegativity, partial charges and chemical reactivity of both molecules. The electron acquiring and electron donating reactive sites for the investigated molecules were found. The electron rich and partially negative charge of the MEP surface is shown in red color; the blue region reveals the electron deficiency and the partially positive charge; the light blue color indicates the slightly electron deficient region; yellow color shows the slightly electron rich region and, the neutral charge/electron area appears in green color. 

**Figure 14:**
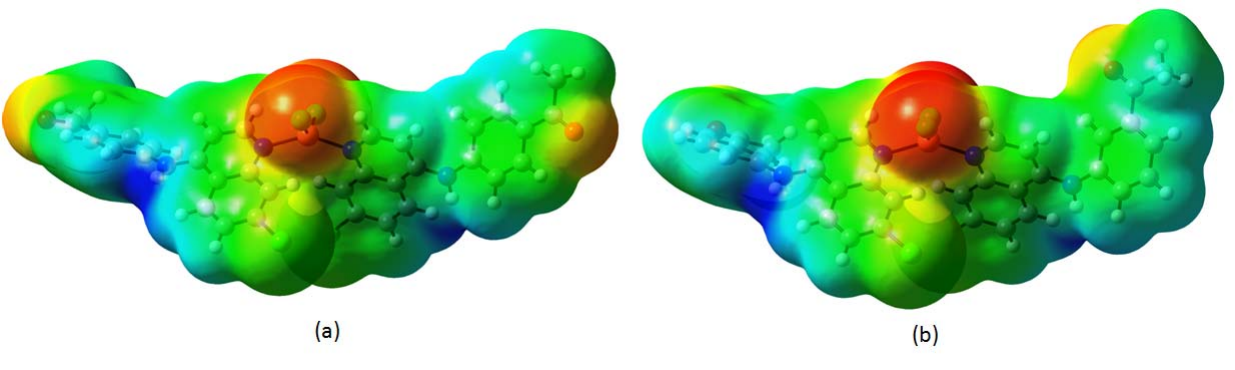
Molecular Electrostatic Potential surface map of the compounds 4a (a) and 5a (b)

It can be seen from Figures 14a and 14b(Fig. 14) that the region around oxygen atoms linked with carbon through double bond and chlorine atoms linked to copper atom specified the most negative potential region (red). The hydrogen atoms attached to the amino group had the maximum concentration of positive charge (blue). The predominance of the green region in the MEP surfaces corresponded to a potential halfway between the two extremes red and dark blue color. In both compounds, total electron density surface mapped with electrostatic potential revealed the presence of high negative charge on the cooper atom while more positive charge around the amino region. The electrostatic potential of the molecules was in the range of - 6.482e-2 (red) to + 6.482e-2 (blue).

### Antimalarial activity

The comparison of biological results on the growth of *P. falciparum*, assays on inhibition of formation of hemozoin/β-hematin and falcipain-2, suggested that several biochemical mechanisms are involved in the antiparasitic action of the compounds evaluated. All the compounds had good activity (˂ 10 μM) against the W2 strain of *P. falciparum* (Table 4(Tab. 4)). The compounds **4** and **5** were shown to be active with IC_50_ values less than 10 μM, being compound **5** the most potent. However, the activity of compound **4** was markedly increased by complexing with Cu(II) metal ion.

Of the two free ligands, the compound **5 **was the best inhibitor, probably due to a stronger interaction with the core of ferriprotoporphyrin IX (FPIX or hematin), which might be mediated by the acetyl group in position 3´ on the phenyl ring. The compound **5** inhibited the parasite growth possibly by acting on the blockade of the polymerization (biomineralization) of hematin to hemozoin, the process of detoxification of free heme by the parasite.

The mechanism that causes the death of the parasite is probably modulated by the interaction of these compounds with the formation of hemozoin. However, it can also be associated with the intraparasitic accumulation of copper in its chelated form, which is very active when it undergoes oxidation-reduction reactions that promote parasite death due to oxidative rupture of the cell membrane.

### Molecular docking studies

The quinoline derivatives are believed to form complexes with dimeric hematin (*β*-hematin), avoiding the formation of hemozoin. This mechanism, as well as the structure-function relationship of established and developing new antimalarial drugs interfering hemozoin formation, have been described extensively. Recently it has been determined that chloroquine (**CQ**) forms complexes with both monomeric and μ-oxo-dimeric FPIX (Leed et al., 2002[24]; de Dios et al., 2003[7]; Gildenhuys et al., 2013[15]; Singh et al., 2014[40]).

**Figure 15:**
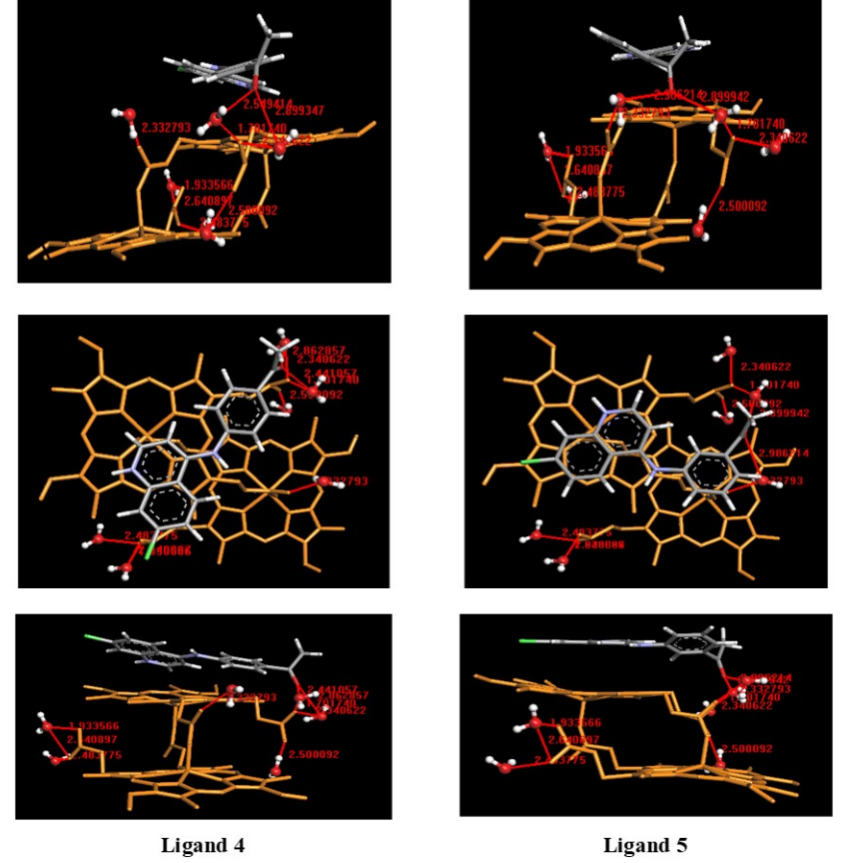
Docking poses of the ligands 4 and 5 in protonated form with hemozoin dimer (*β*-hematin) solvated

Therefore, we decided to evaluate the mode of interaction of free ligands (**4**,** 5**) in the protonated form with hemozoin dimer (*β*-hematin) solvated, in order to inquire about the difference of both ligands in the inhibition of hemozoin formation. For this purpose, molecular docking of free ligands in the protonated form with *β*-hematin solvated as the binding site was performed, using ArgusLab 4.0.1. As shown in Figure 15(Fig. 15), the carbonyl groups of both ligands formed hydrogen bonds with the free propionic acid functional groups of the dimer, using water molecules as an interaction bridge, which could prevent possible interactions with another dimer and avoid the growth of the hemozoin polymer. 

Additionally, ligand **5** showed an interaction with the propionate coordinated to Fe(III), mediated by a water molecule. However, the marked difference of both ligands in the ability to inhibit hemozoin formation seemed to be related to the establishment of favorable π-π interactions. The quinoline ring in ligand **5 **was oriented toward the planar and electron-rich periphery region of the iron-porphyrin system, thus establishing effective π-π interaction. While ligand **4 **showed a pose, which does not favor such interaction, explaining the observed decrease of activity. This analysis seems to support the hypothesis that π-π interaction between quinoline derivatives and the electronic system of hematin governed the formation of adducts (Kumar et al., 2007[23]; Webster et al., 2009[45]).

## Conclusions

In this report, we present the results of spectroscopic, spectrometric studies and antimalarial activity carried out on two types of [(7-chloroquinolin-4-yl)amino]acetophenones ligands and its corresponding mono-chelated complexes. Also, DFT studies and molecular docking were performed to complement experimental studies of structural characterization and biological functionality. 

A detailed investigation on the gas phase chemistry of the protonated compounds using CID mass spectrometry, allowed to identify and follow the changes in structural populations for better understand their possible dissociation pathways. The characterization of the fragmentation pattern of protonated free ligands was extended here to fragments as low as *m/z* 43, while for coordination complexes it extended to fragments at *m/z* 80 and *m/z* 111. Almost all the high and low *m/z,* as well as the high and low abundance fragments, were identified and, dissociation pathways were proposed to form them. We have shown that CID of protonated [(7-chloroquinolin-4-yl)amino]acetophenones ligands (**4** and **5**) generated a dominant product ion at *m/z* 253-254 through two probable routes, one involving an intermediate of neutral/ion complex type that undergoes an intermolecular electrophilic reaction; and the other more direct one that involves the consecutive release of methane and carbon monoxide. Both routes contributed to this ion to be the most abundant. 

In the case of the complexes, the experiments reveal an oxidation state 1+ for the metal, these suggest that copper undergoes a reduction of Cu(II) → Cu(I) during ionization by electrospray; the main pathway of fragmentation involves the loss of a ligand molecule in its free form, dissociation promoted perhaps by steric repulsions; generating the dominant product ion at *m/z* 359 (^63^Cu) - 361 (^65^Cu). 

All the spectral results suggest that the ligand acts as chelating species coordinating the metal through the endocyclic nitrogen of the quinoline ring in both complexes, with general formulae expressed in two ways, according to the phase in which they are: [Cu(L)_2_Cl_2_] for the solid phase and [Cu(L)_2_][2Cl] for the liquid phase. The EPR study of the Cu(II) complexes indicates a probable distorted tetrahedral coordination geometry. This result confirmed by calculated optimized structures at the DFT/B3LYP method with the 6-31G (d,p) basis set. 

The analysis of frontier molecular orbitals of the complexes, with HOMO-LUMO energy gap of 3.847 eV (**4a**) and 3.932 eV (**5a**), is interpreted as with high chemical activity, which can explain the good biological activity shown on the inhibition of the growth of *P. falciparum*. In general, the activity of compounds **4** and **5** was increased by complexing with the Cu(II) metal ion. The molecular docking study carried out to understand the reason for the biological activity of free ligands, showed the participation of water molecules as an interaction bridge through hydrogen bonds between free ligands (**4**, **5**) and *β*-hematin; at the same time, more efficient π-π interactions were observed between **5** and the electronic hematin system. This analysis seems to support the hypothesis that π-π interaction between quinoline derivatives and the electronic system of hematin governs the formation of adducts.

## Funding

This research was supported by the Formative Research Unit of Research Directorate of Central University of Ecuador, under grant N° CIF4-CE-FIG-3.

## Acknowledgements

The authors sincerely thank Prof. Delfín Moronta, from the Laboratory of Paramagnetism, School of Physics, Faculty of Sciences, Central University of Venezuela, for his help in EPR studies. The authors also appreciate the assistance in the ESI-CID-MS^2 ^studies to Ana Y. Angarita H., from the Mass Spectrometry Laboratory, Chemistry Center, Venezuelan Institute of Scientific Research (IVIC), and to Dr. Anibal Sierralta, Laboratory of Physical Chemistry and Computational Catalysis, Venezuelan Institute of Scientific Research for his valuable collaboration in the use and management of structural calculation programs.

## Declaration of conflicting interests

The authors declare no potential conflicts of interest with respect to the research, authorship, and/or publication of this article.

## Appendix A. Supplementary material

Supplementary data associated with this article can be found in a seperate file.

## Supplementary Material

Supplementary material

## Figures and Tables

**Table 1:**
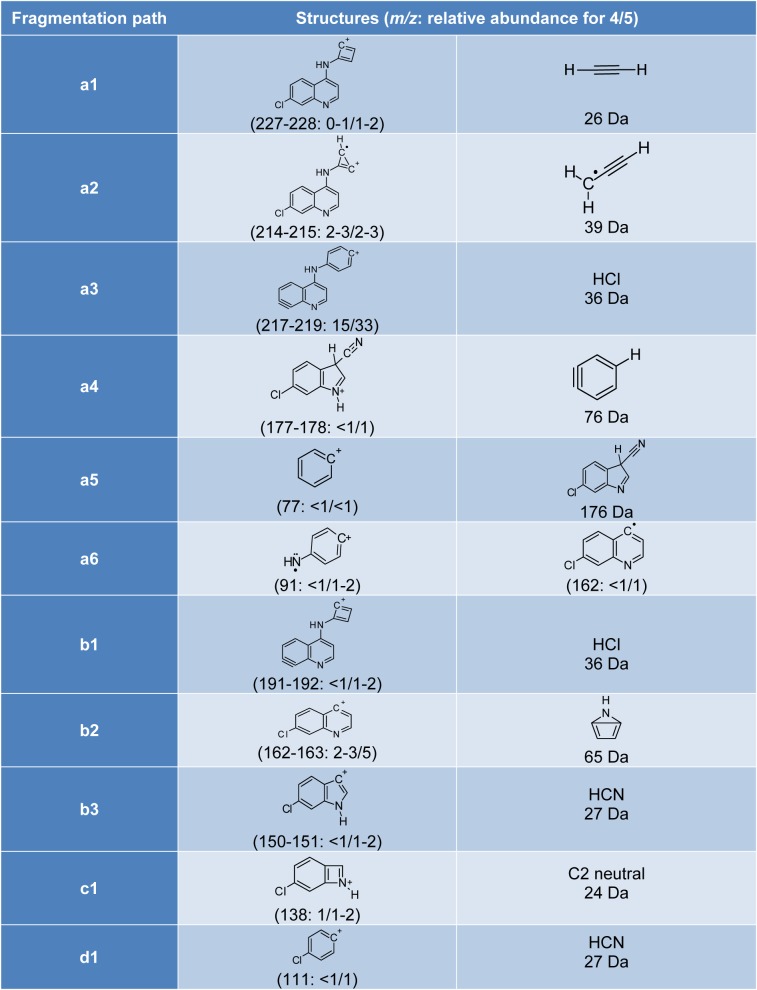
Proposed structures for some fragments originating from the ion at *m/z* 253-254

**Table 2:**
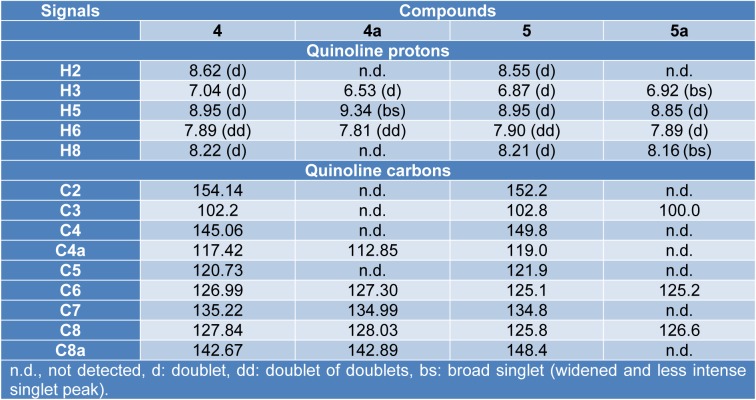
Comparison of NMR signals of the quinoline ring between ligands (4, 5) and complexes (4a, 5a)

**Table 3:**
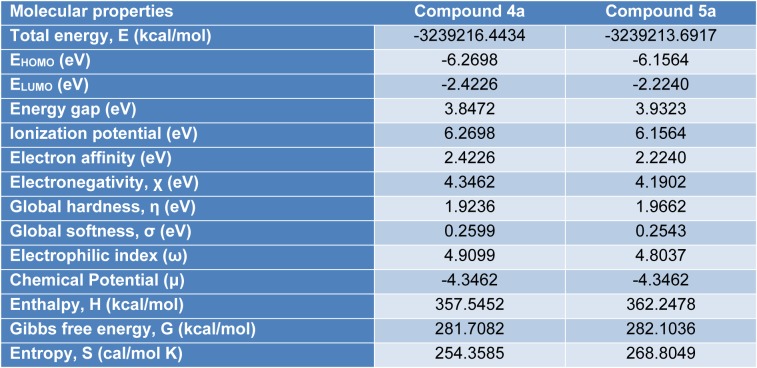
Thermodynamic parameters of the compounds 4a and 5a

**Table 4:**
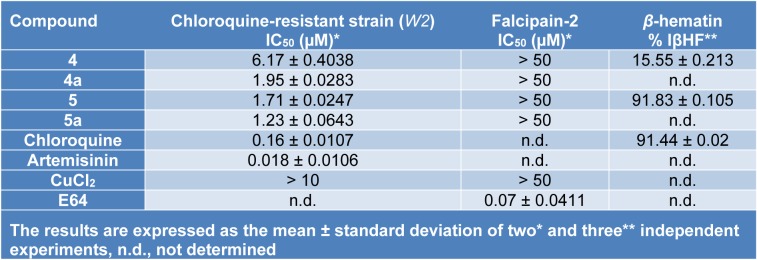
*In vitro* activity results for synthesized compounds against growth of *P. falciparum*, Falcipain-2 and hemozoin formation

## References

[1] Angelusiu MV, Almăjan GL, Ilies DC, Rosu T, Negoiu M (2008). Cu(II) complexes with nitrogen-oxygen donor ligands: synthesis and biological activity. Chem Bull "POLITEHNICA" Univ. (Timisoara).

[2] Baelmans R, Deharo E, Muñoz V, Sauvain M, Ginsburg H (2000). Experimental conditions for testing the inhibitory activity of chloroquine on the formation of beta-hematin. J Exp Parasitol.

[3] Bahl D, Athar F, Pereira M, Santos M, Magalhaes D, Mohan R (2010). Structure–activity relationships of mononuclear metal–thiosemicarbazone complexes endowed with potent antiplasmodial and antiamoebic activities. Bioorg Med Chem.

[4] Camacho J, Ferrer R, Maldonado A, Navarro M (2005). Química de coordinación como un arma para el desarrollo de potenciales drogas antimaláricas. Estudio de los posibles mecanismos de acción. Rev Facult Farm Univ Centr Venezuela.

[5] Chipeleme A, Gut J, Rosenthal PJ, Chibale K (2007). Synthesis and biological evaluation of phenolic. Mannich bases of benzaldehyde and (thio) semicarbazone derivatives against the cysteine protease falcipain-2 and a chloroquine resistant strain of Plasmodium falciparum. Bioorg Med Chem.

[6] Chiyanzu I, Clarkson C, Smith PJ, Lehman J, Gut J, Rosenthal PJ (2005). Design, synthesis and anti-plasmodial evaluation in vitro of new 4-aminoquinoline isatin derivatives. Bioorg Med Chem.

[7] de Dios AC, Tycko R, Ursos LMB, Roepe PD (2003). NMR studies of chloroquine−ferriprotoporphyrin IX complex. J Phys Chem A.

[8] Demarque DP, Crotti AEM, Vessecchi R, Lopes JLC, Lopes NP (2016). Fragmentation reactions using electrospray ionization mass spectrometry: an important tool for the structural elucidation and characterization of synthetic and natural products. Nat Prod Rep.

[9] Dennington R, Keith T, Millam J (2007). GaussView, Version 4.1.2. Semichem Inc.

[10] Egan TJ, Hunter R, Kaschula CH, Marques HM, Misplon A, Walden J (2000). Structure−function relationships in aminoquinolines:  effect of amino and chloro groups on quinoline−hematin complex formation, inhibition of β-hematin formation, and antiplasmodial activity. J Med Chem.

[11] Ferrer R, Lobo G, Gamboa N, Rodrigues J, Abramjuk C, Jung K (2009). Synthesis of [(7-Chloroquinolin-4-yl)amino]chalcones: potential antimalarial and anticancer agents. Sci Pharm.

[12] Filges U, Grützmacher H-Fr (1987). Fragmentations of protonated acetophenones via intermediate ion–molecule complexes. Org Mass Spectrom.

[13] Foresman JB, Frisch A (1996). Exploring chemistry with electronic structure methods.

[14] Frisch MJ, Trucks GW, Schlegel HB, Scuseria GE, Robb MA, Cheeseman JR (2009). Gaussian 09.

[15] Gildenhuys J, le Roex T, Egan TJ, de Villiers KA (2013). The single crystal X-ray structure of β-hematin DMSO solvate grown in the presence of chloroquine, a β-hematin growth-rate inhibitor. J Am Chem Soc.

[16] Hathaway BJ, Billing DE (1970). The electronic properties and stereochemistry of mono-nuclear complexes of the copper(II) ion. Coord Chem Rev.

[17] Haynes WM (2012). CRC Handbook of chemistry and physics.

[18] Hoffmann SK, Hilczer W, Goslar J (1996). EPR, electron spin–lattice relaxation, and debye temperature of Cu(II)-doped triglycine selenate crystal. J Magn Reson Series A.

[19] Hunter EPL, Lias SG (1998). Evaluated gas phase basicities and proton affinities of molecules: an update. J Phys Chem Ref Data.

[20] Kimura K, Katsumata S, Achiba Y, Yamazaki T, Iwata S (1981). Handbook of HeI photoelectron spectra of fundamental organic molecules: ionization energies, ab initio assignments, and valence electronic structure for 200 molecules.

[21] Kouznetsov V, Amado D (2008). Antimalarials: construction of molecular hybrids based on chloroquine. Univ Scientiarum.

[22] Kouznetsov V, Gómez-Barrio A (2009). Recent developments in the design and synthesis of hybrid molecules based on aminoquinoline ring and their antiplasmodial evaluation. Eur J Med Chem.

[23] Kumar S, Guha M, Choubey V, Maity P, Bandyopadhyay U (2007). Antimalarial drugs inhibiting hemozoin (β-hematin) formation: A mechanistic update. Life Sci.

[24] Leed A, DuBay K, Sears D, de Dios AC, Roepe PD (2002). Solution structures of antimalarial drug−heme complexes. Biochemistry.

[25] Murphy B, Hathaway B (2003). The stereochemistry of the copper(II) ion in the solid-state/some recent perspectives linking the Jahn Teller effect, vibronic coupling, structure correlation analysis, structural pathways and comparative X-ray crystallography. Coord Chem Rev.

[26] Murray JS, Sen K (1996). Molecular electrostatic potentials concepts and applications.

[27] Pagola S, Stephens PW, Bohle DS, Kosar AD, Madsen SK (2000). The structure of malaria pigment β-haematin. Nature.

[28] Park S-G, Cui S, Jo S-C, Nam S, Lee Y-I (2007). Structural characterization of alachlor complexes with transition metal ions by electrospray ionization tandem mass spectrometry. Microchem J.

[29] Parr RJ, Szentpály LV, Liu S (1999). Electrophilicity index. J Am Chem Soc.

[30] Parra YJ, Ferrer RE (2012). Interdisciplinarity in the rational design of enzyme inhibitory antimalarial drugs. Informe Médico.

[31] Pearson RG (1989). Absolute electronegativity and hardness: applications to organic chemistry. J Org Chem.

[32] Pengyuan L, Nan H, Yuanjiang P, Yaping T (2010). Ion-neutral complexes resulting from dissociative protonation: fragmentation of α-furanylmethyl benzyl ethers and 4-N,N-dimethylbenzyl benzyl ethers. J Am Soc Mass Spectrom.

[33] Pirnau A, Chis V, Oniga O, Leopold N, Szabo L, Baias M (2008). Vibrational and DFT study of 5-(3-pyridyl-methylidene)-thiazolidine-2-thione-4-one. Vib Spectrosc.

[34] Rasoloson D, Shi L, Chong C, Kafsack B, Sullivan D (2004). Copper pathways in Plasmodium falciparum infected erythrocytes indicate an efflux role for the copper P-ATPase. Biochem J.

[35] Reed AE, Curtiss LA, Weinhold F (1988). Intermolecular interactions from a natural bond orbital, donor-acceptor viewpoint. Chem Rev.

[36] Reinders PP, Van Vianen PH, Van Der Keur M, Van Engen A, Janse CJ, Tanke HJ (1995). Computer software for testing drug susceptibility of malaria parasites. Cytometry.

[37] Rosenthal PJ, Olson JE, Lee GK, Palmer JT, Klaus JL, Rasnick D (1996). Antimalarial effects of vinyl sulfone cysteine protease inhibitors. Antimicrob Agents Chemother.

[38] Shenai BR, Sijwali PS, Singh A, Rosenthal PJ (2000). Characterization of native and recombinant Falcipain-2, a principal trophozoite cysteine protease and essential hemoglobinase of Plasmodium falciparum. J Biol Chem.

[39] Shi T, Siu KW, Hopkinson AC (2006). Fragmentation of doubly charged metal–acetamide complexes: Second ionization energies and dissociation chemistries. Int J Mass Spectrom.

[40] Singh K, Kaur H, Smith P, de Kock C, Chibale K, Balzarini J (2014). Quinoline–pyrimidine hybrids: synthesis, antiplasmodial activity, SAR, and mode of action studies. J Med Chem.

[41] Sreekanth A, Prathapachandra Kurup MR (2003). Structural and spectral studies on four coordinate copper(II) complexes of 2-benzoylpyridine N(4), N(4)- (butane-1,4-diyl)thiosemicarbazone. Polyhedron.

[42] Tao W, Klemm RB, Nesbitt FL, Stief JL (1992). A discharge flow-photoionization mass spectrometric study of hydroxymethyl radicals (H2COH and H2COD): Photoionization spectrum and ionization energy. J Phys Chem.

[43] Thielking G, Filges U, Grützmacher H-Fr (1992). Remote fragmentations of protonated aromatic carbonyl compounds via internal reactions in intermediary ion-neutral complexes. J Am Soc Mass Spectrom.

[44] Underwood CC, Stadelman BS, Sleeper ML, Brumaghim JL (2013). Synthesis and electrochemical characterization of [Ru(NCCH3)6]2+, tris(acetonitrile) tris(pyrazolyl)borate, and tris(acetonitrile) tris(pyrazolyl)methane ruthenium(II) complexes. Inorg Chim Acta.

[45] Webster GT, McNaughton D, Wood BR (2009). Aggregated enhanced Raman scattering in Fe(III)PPIX solutions: the effects of concentration and chloroquine on excitonic interactions. J Phys Chem B.

[46] Wenthold PG, Liu X (2001). Competing mechanisms for methyl cation formation upon collision-induced dissociation of protonated acetaldehyde. Int J Mass Spectrom.

[47] WHO (2018). World malaria report 2018.

[48] Wikstrom JP, Filatov AS, Staples RJ, Guifarro CR, Rybak-Akimova EV (2010). Steric and counterion effects on the structure of dipicolylamine nickel complexes. Inorg Chim Acta.

[49] Ya-Ping T (2012). Dissociative protonation and fragmentation: Retro-Friedel–Crafts reactions of heterocyclic drug and metabolite molecules in mass spectrometry. Int J Mass Spectrom.

[50] Ya-Ping T (2006). Dissociative protonation sites:  reactive centers in protonated molecules leading to fragmentation in mass spectrometry. J Org Chem.

[51] Young DC (2001). Computational chemistry: A practical guide for applying techniques to real world problems.

[52] Zhidomirov GM, Salikhov KM, Raitsimring AM, Tsvetkov YD (1969). The nature of the broadening of EPR lines as determined by dipole-dipole interaction in magnetically diluted systems. J Struct Chem.

