# Supplementary material for: [(7-chloroquinolin-4-yl)amino]acetophenones and their copper(II) derivatives: Synthesis, characterization, computational studies and antimalarial activity
Source: EXCLI J. 2019 Oct 28;18:962–87. doi: 10.17179/excli2019-1805 (PMC6868921; doi:10.17179/excli2019-1805)
Supplement: Supplementary material [file EXCLI-18-962-s-001.pdf]

EXCLI Journal 2019;18:962-987 – ISSN 1611-2156
Received: September 11, 2019, accepted: October 17, 2019, published: October 28, 2019

Supplementary material to:

[(7-CHLOROQUINOLIN-4-YL)AMINO]ACETOPHENONES AND THEIR COPPER(II) DERIVATIVES: SYNTHESIS, CHARACTERIZATION, COMPUTATIONAL STUDIES AND ANTIMALARIAL ACTIVITY

Yonathan de J. Parra[a*], Felix D. Andueza L[a], Rosa E. Ferrer M[b], Julia Bruno Colmenarez[c], María E. Acosta[d], Jaime Charris[e], Philip J. Rosenthal[f], Jiri Gut[f]

[a] Escuela de Ingeniería Ambiental, Facultad de Ingeniería en Geología, Minas, Petróleos y Ambiental, Universidad Central del Ecuador, Quito 170521, Ecuador
[b] Departamento de Química, Facultad de Humanidades y Educación, Universidad del Zulia, Apartado 526, Maracaibo, Estado Zulia, Venezuela
[c] Centro de Investigación y Tecnología de Materiales (CITeMa), Instituto Venezolano de Investigaciones Científicas, Apartado 20632, Altos de Pipe 1020-A Estado Miranda, Venezuela
[d] Laboratorio de Bioquímica, Facultad de Farmacia, Universidad Central de Venezuela, Apartado 47206, Los Chaguaramos, 1041-A Caracas, Venezuela
[e] Laboratorio de Síntesis Orgánica, Facultad de Farmacia, Universidad Central de Venezuela, Apartado 47206, Los Chaguaramos, 1041-A Caracas, Venezuela
[f] Department of Medicine, University of California, Box 0811, San Francisco, California 94143, USA

* **Corresponding author:** Prof. Yonathan de J. Parra, Escuela de Ingeniería Ambiental, Facultad de Ingeniería en Geología, Minas, Petróleos y Ambiental, Universidad Central del Ecuador, Quito 170521, Ecuador; Tel: +593-2-2550588, ext. 111; E-mail: ydparra@uce.edu.ec

http://dx.doi.org/10.17179/excli2019-1805

This is an Open Access article distributed under the terms of the Creative Commons Attribution License (http://creativecommons.org/licenses/by/4.0/).

EXCLI Journal 2019;18:962-987 – ISSN 1611-2156
Received: September 11, 2019, accepted: October 17, 2019, published: October 28, 2019

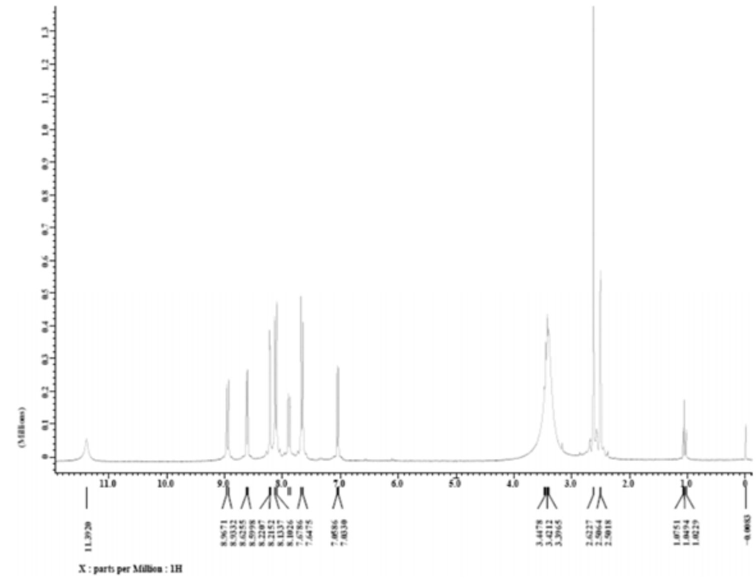

Supplementary Figure 1: ^{1}H NMR spectrum of 4-[(7-chloroquinolin-4-yl)amino]acetophenone (**4**)

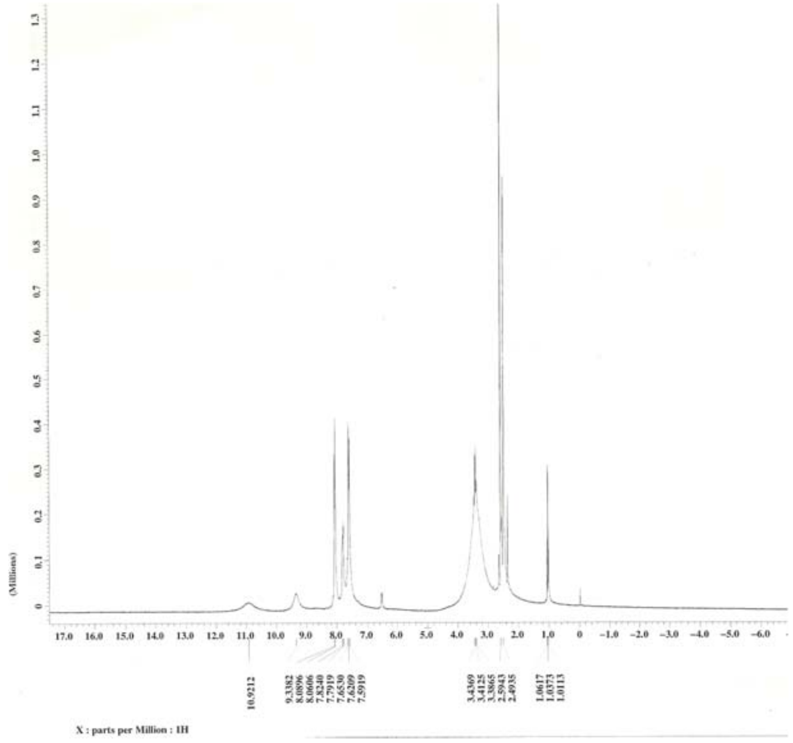

Supplementary Figure 2: ^{1}H NMR spectrum of {4-[(7-chloroquinolin-4-yl)amino]acetophenone} copper (II) chloride (**4a**)

EXCLI Journal 2019;18:962-987 – ISSN 1611-2156
Received: September 11, 2019, accepted: October 17, 2019, published: October 28, 2019

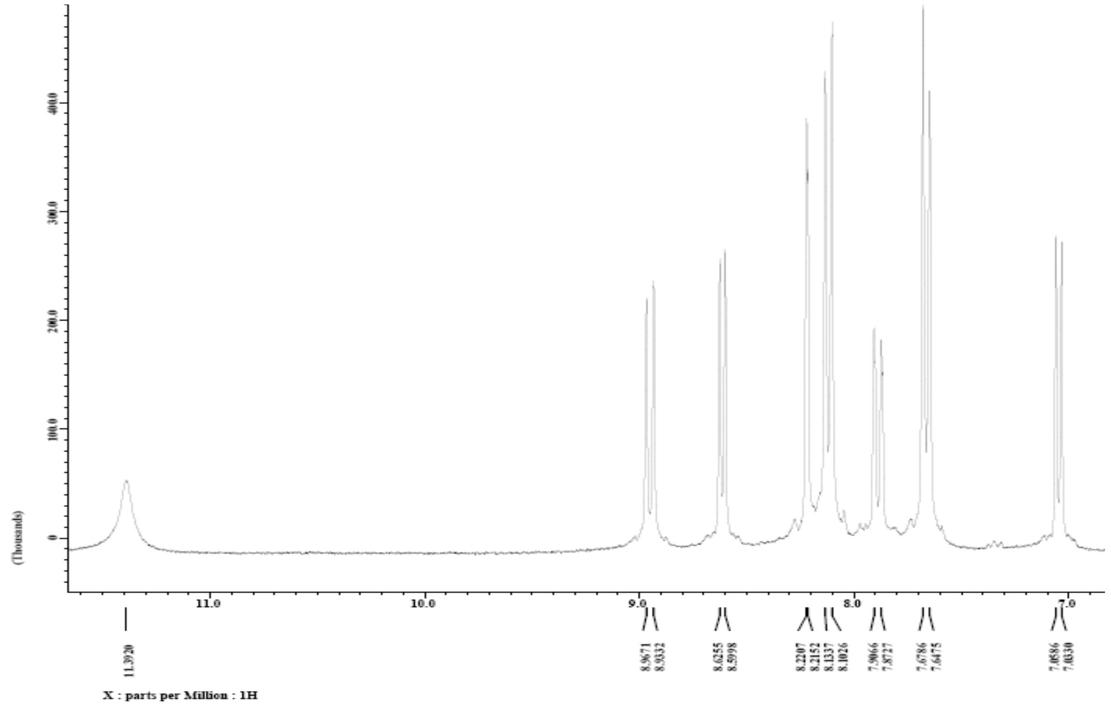

Supplementary Figure 3: Amplified ^{1}H NMR spectrum of 4-[(7-chloroquinolin-4-yl)amino]acetophenone (**4**)

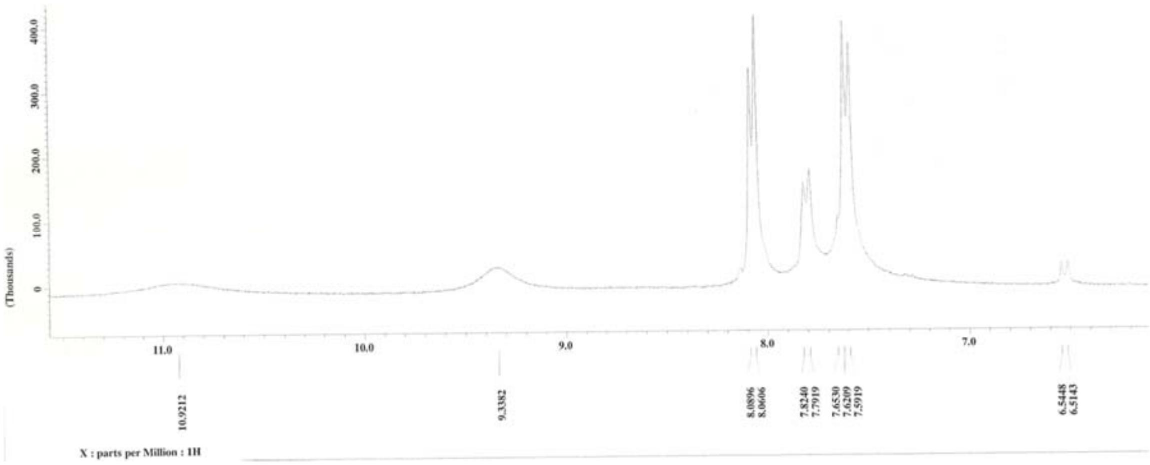

Supplementary Figure 4: Amplified ^{1}H NMR spectrum of {4-[(7-chloroquinolin-4-yl)amino]acetophenone}copper(II) chloride (**4a**)

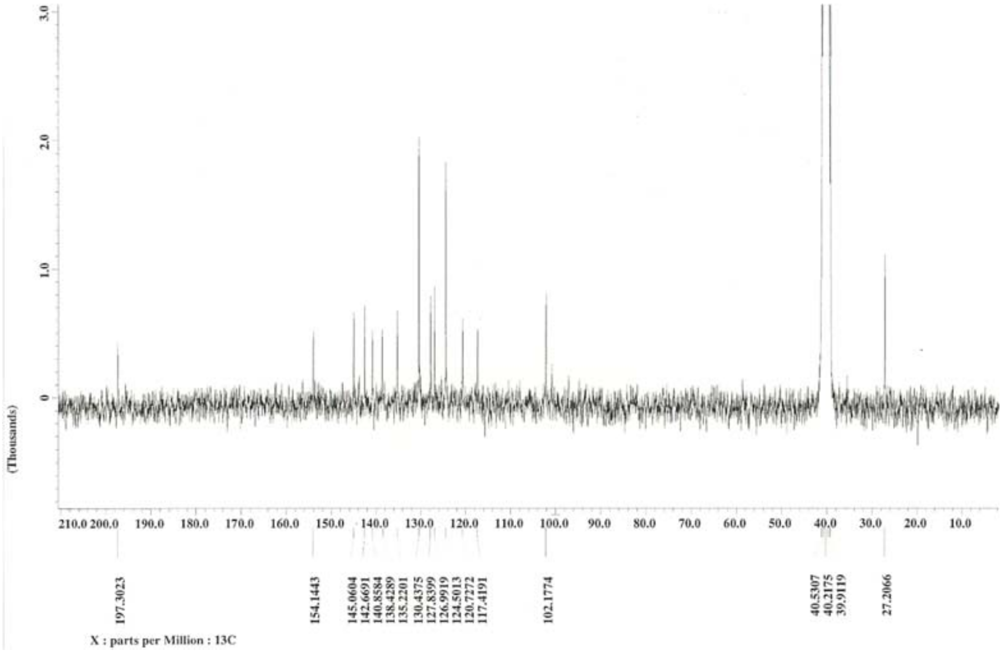

Supplementary Figure 5: ^{13}C NMR spectrum of 4-[(7-chloroquinolin-4-yl)amino]acetophenone (**4**)

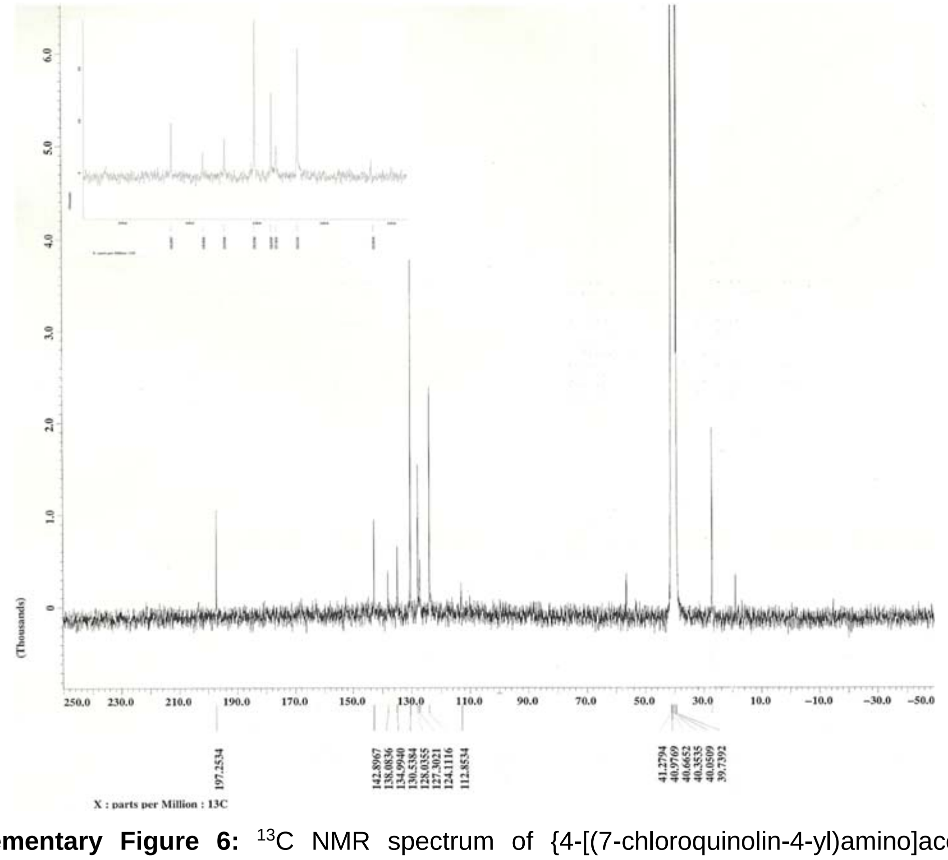

Supplementary Figure 6: ^{13}C NMR spectrum of {4-[(7-chloroquinolin-4-yl)amino]acetophenone}copper(II) chloride (**4a**)

EXCLI Journal 2019;18:962-987 – ISSN 1611-2156
Received: September 11, 2019, accepted: October 17, 2019, published: October 28, 2019

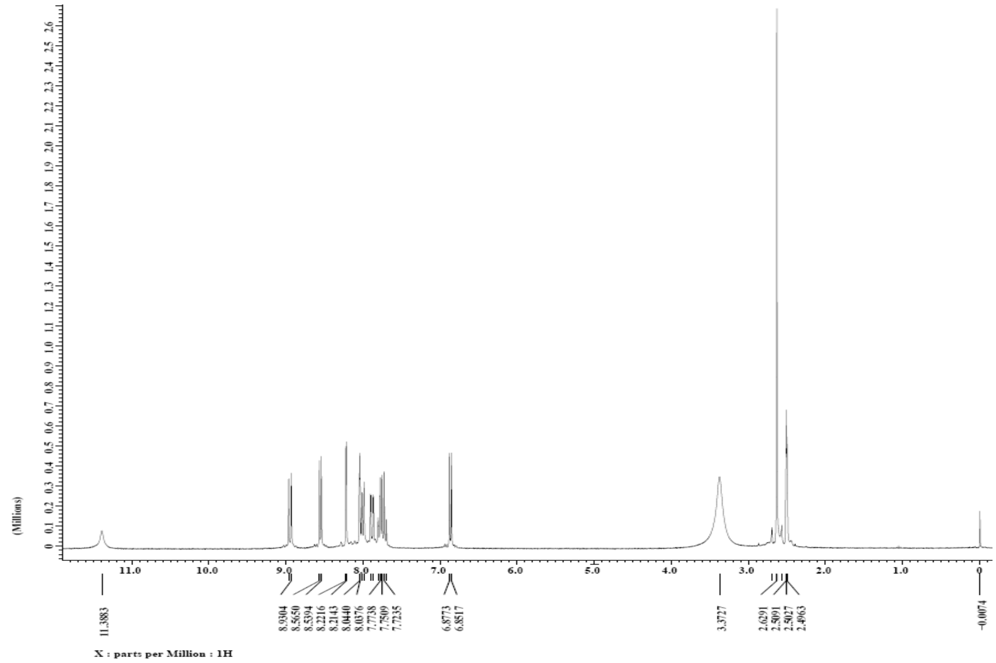

Supplementary Figure 7: ^{1}H NMR spectrum of 3-[(7-chloroquinolin-4-yl)amino]acetophenone (**5**)

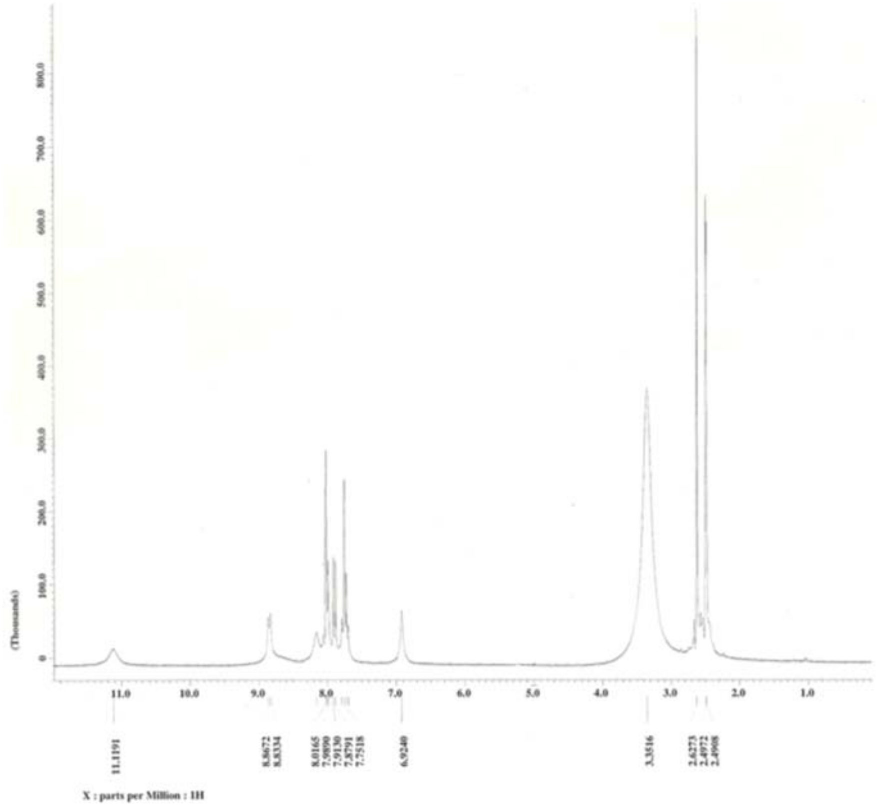

Supplementary Figure 8: ^{1}H NMR spectrum of {3-[(7-chloroquinolin-4-yl)amino]acetophenone} copper(II) chloride (**5a**)

EXCLI Journal 2019;18:962-987 – ISSN 1611-2156
Received: September 11, 2019, accepted: October 17, 2019, published: October 28, 2019

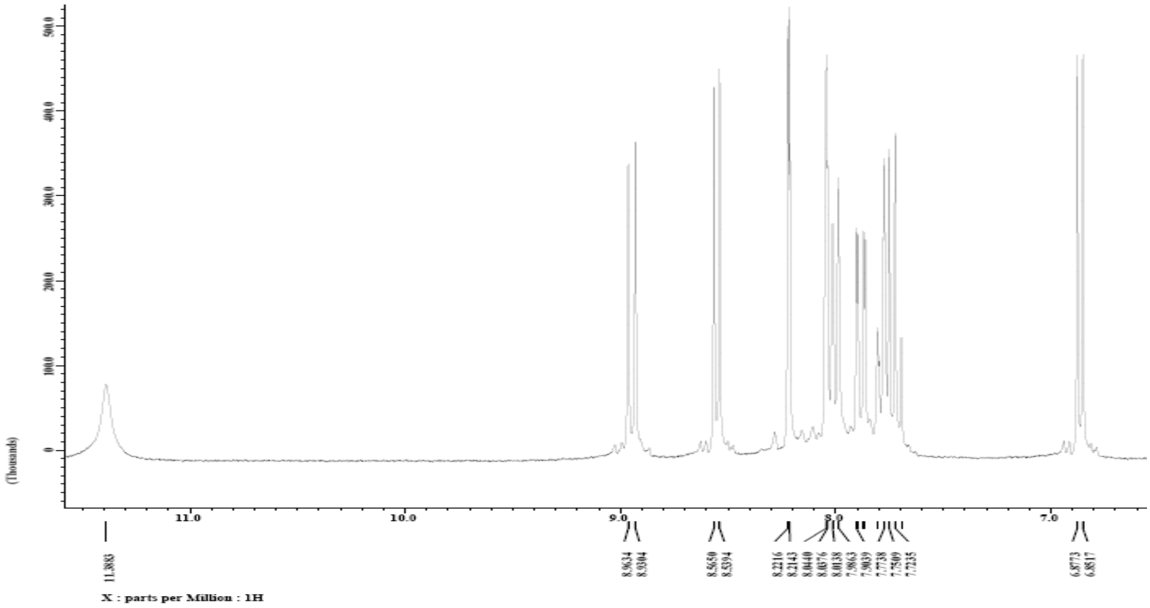

Supplementary Figure 9: Amplified ^{1}H NMR spectrum of 3-[(7-chloroquinolin-4-yl)amino]acetophenone (**5**)

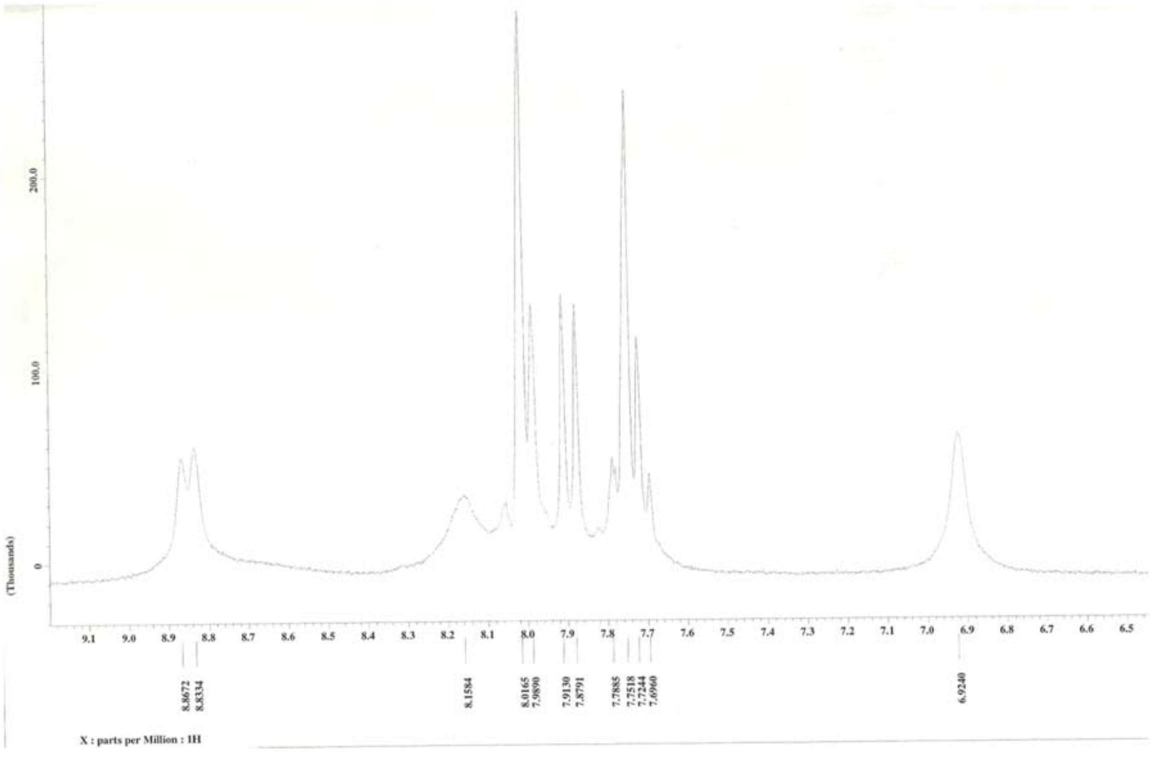

Supplementary Figure 10: Amplified ^{1}H NMR spectrum of {3-[(7-chloroquinolin-4-yl)amino]acetophenone}copper(II) chloride (**5a**)

EXCLI Journal 2019;18:962-987 – ISSN 1611-2156
Received: September 11, 2019, accepted: October 17, 2019, published: October 28, 2019

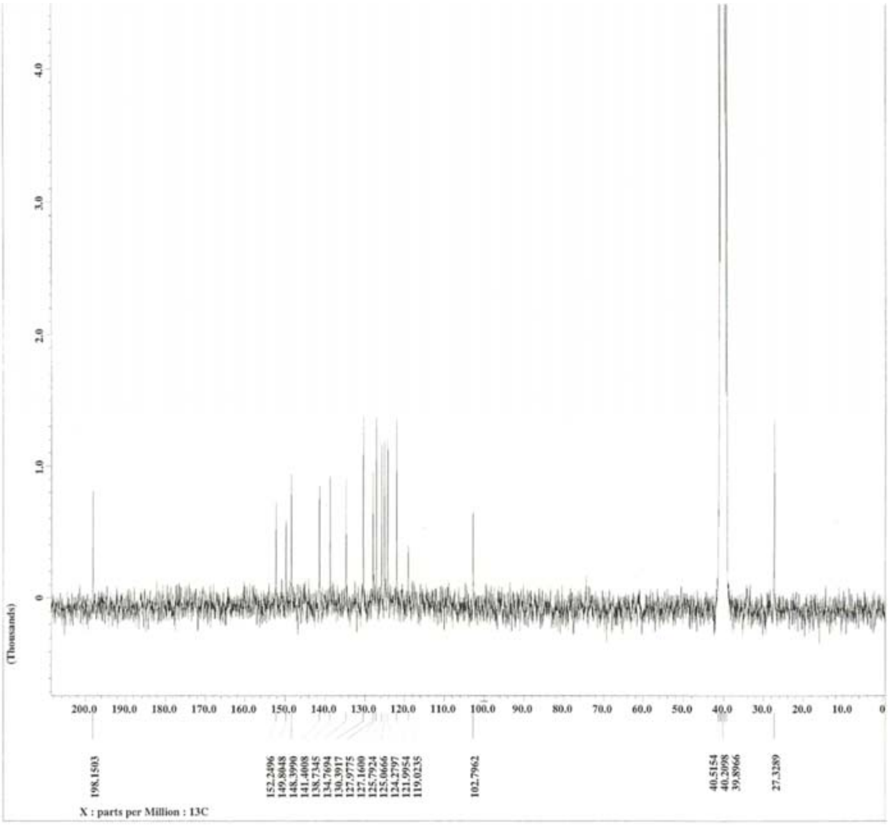

Supplementary Figure 11: ^{13}C NMR spectrum of 3-[(7-chloroquinolin-4-yl)amino]acetophenone (**5**)

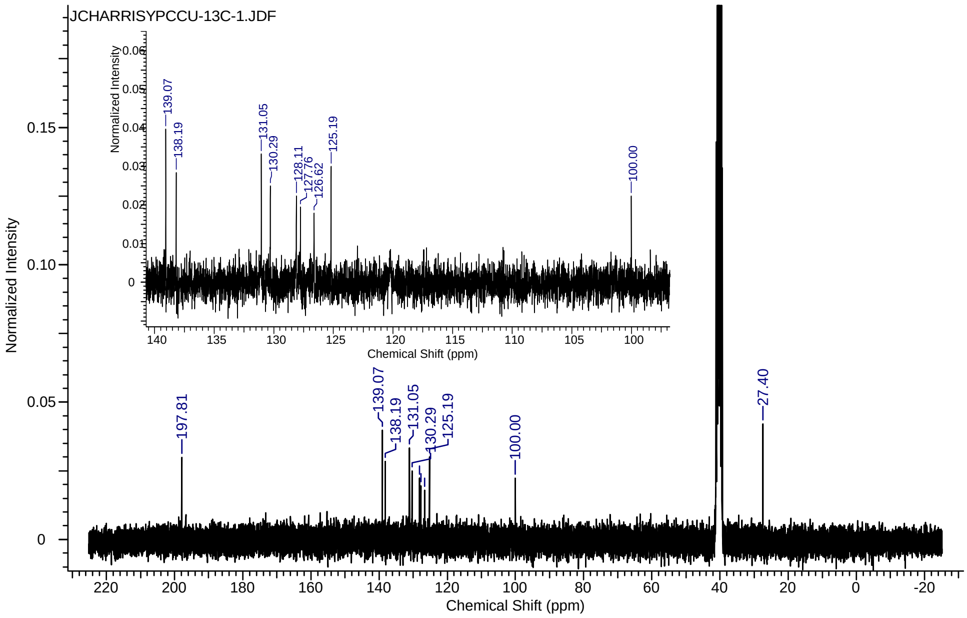

Supplementary Figure 12: ^{13}C NMR spectrum of {3-[(7-chloroquinolin-4-yl)amino]acetophenone}copper(II) chloride (**5a**)

EXCLI Journal 2019;18:962-987 – ISSN 1611-2156
Received: September 11, 2019, accepted: October 17, 2019, published: October 28, 2019

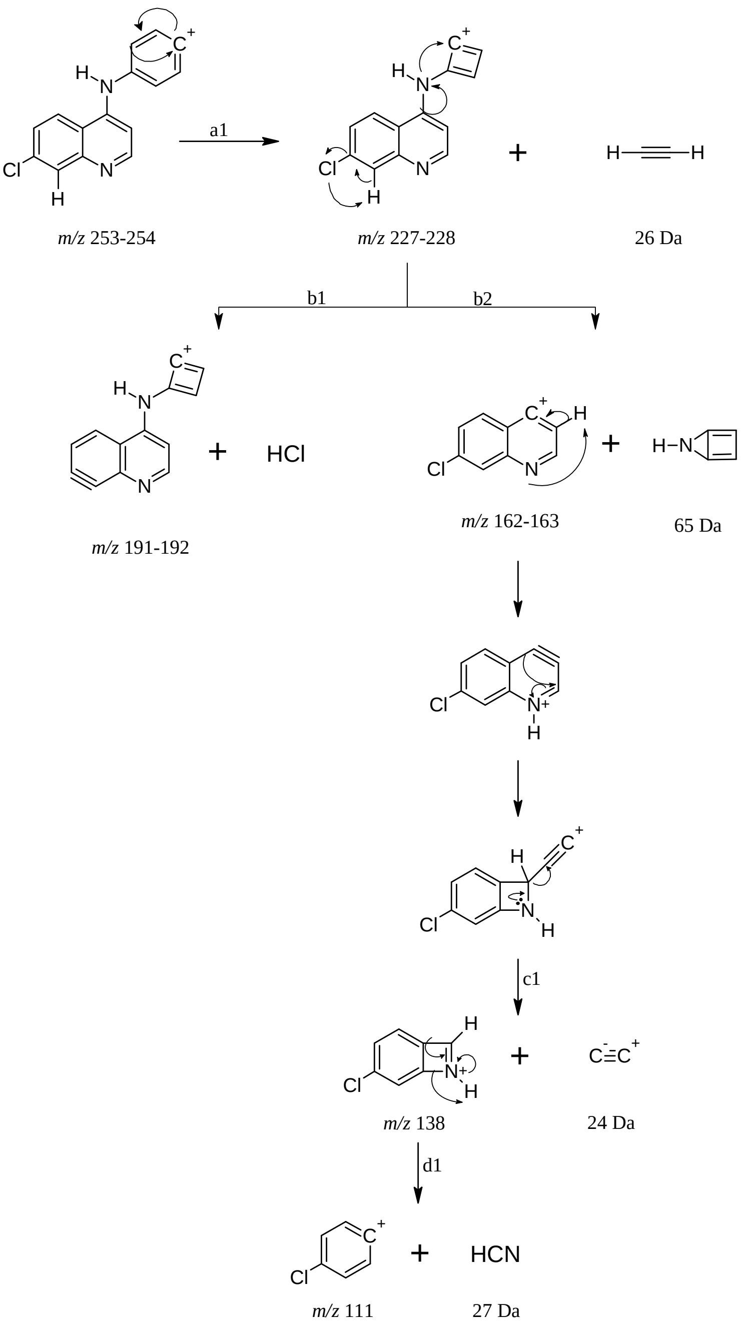

Supplementary Figure 13: Dissociative heterolytic mechanism for the a1, b1, b2, c1, d1 pathways; from the fragment at *m/z* 253-254 generated by the precursor molecular ion at *m/z* 297 for compound **4**. (Analogue mechanism for compound **5**)

EXCLI Journal 2019;18:962-987 – ISSN 1611-2156
Received: September 11, 2019, accepted: October 17, 2019, published: October 28, 2019

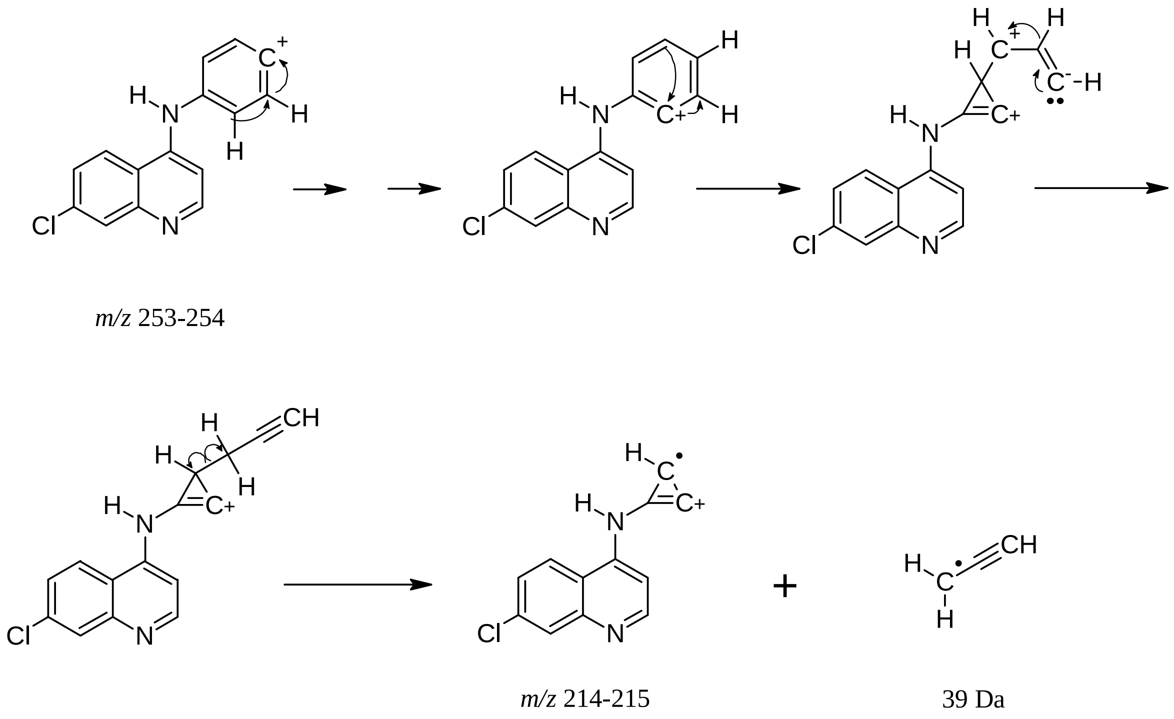

Supplementary Figure 14: Dissociative homolytic mechanism for a2 pathway

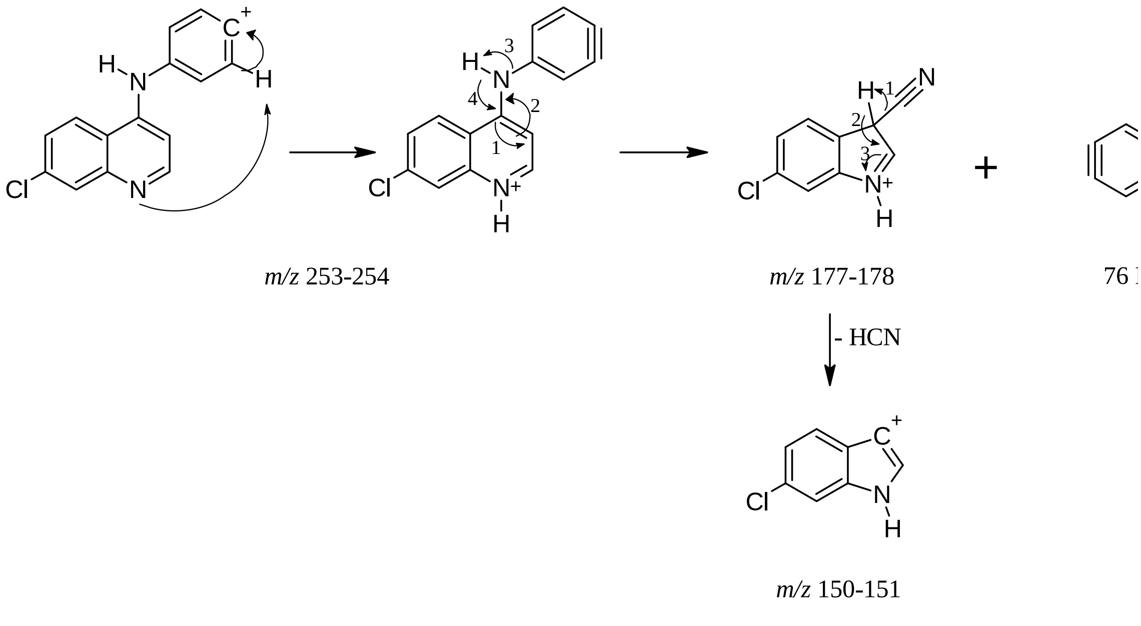

Supplementary Figure 15: Dissociative heterolytic mechanism for the a4 and b3 pathways

EXCLI Journal 2019;18:962-987 – ISSN 1611-2156
Received: September 11, 2019, accepted: October 17, 2019, published: October 28, 2019

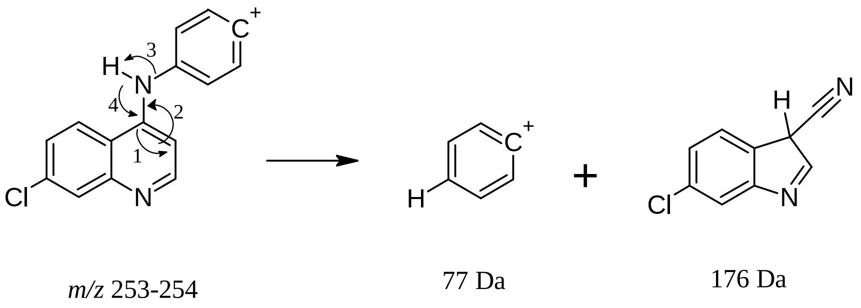

Supplementary Figure 16: Dissociative heterolytic mechanism for the a5 pathway

EXCLI Journal 2019;18:962-987 – ISSN 1611-2156
Received: September 11, 2019, accepted: October 17, 2019, published: October 28, 2019

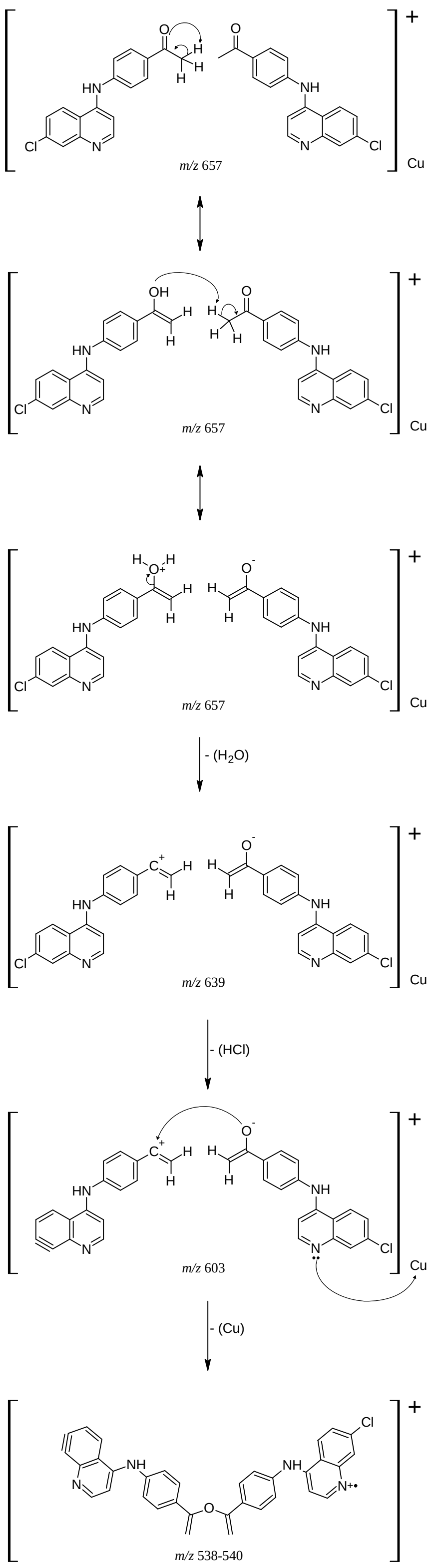

Supplementary Figure 17: Minor fragmentation channel of the cationic fragment [**4a**]$^+$ at *m/z* 657

EXCLI Journal 2019;18:962-987 – ISSN 1611-2156
Received: September 11, 2019, accepted: October 17, 2019, published: October 28, 2019

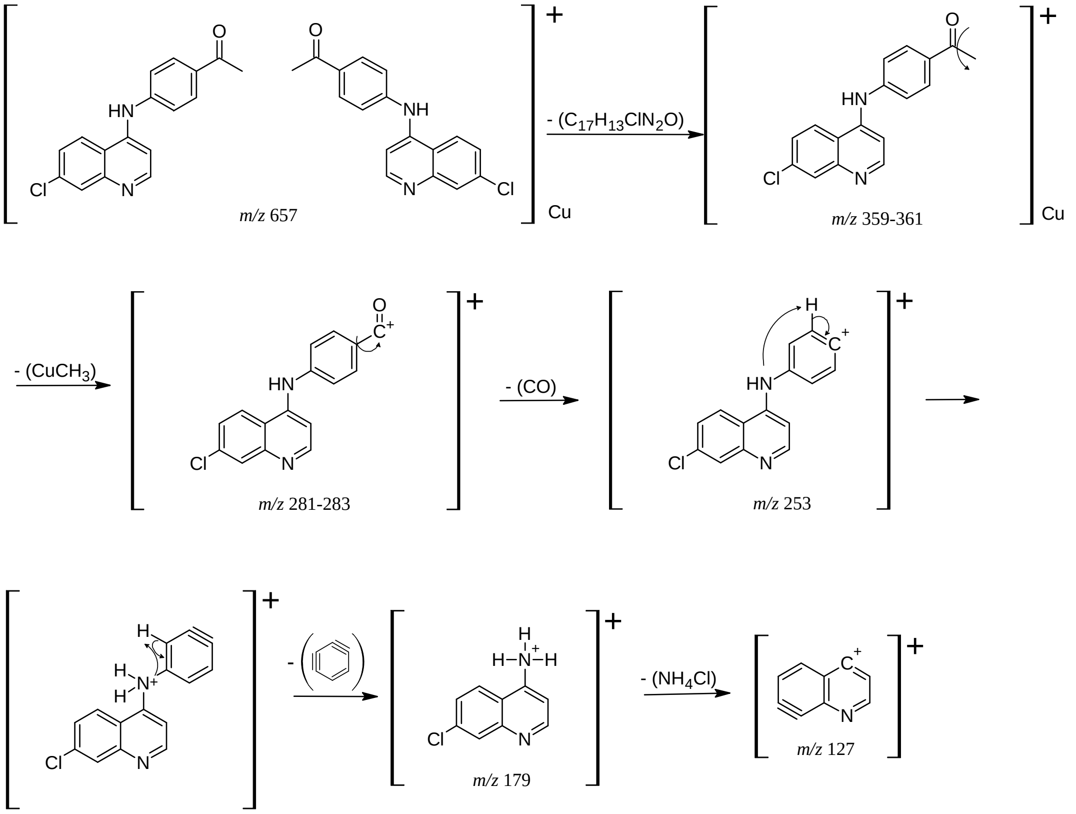

Supplementary Figure 18: Extensive dissociation of the cationic fragment [**4a**]$^+$ at *m/z* 657

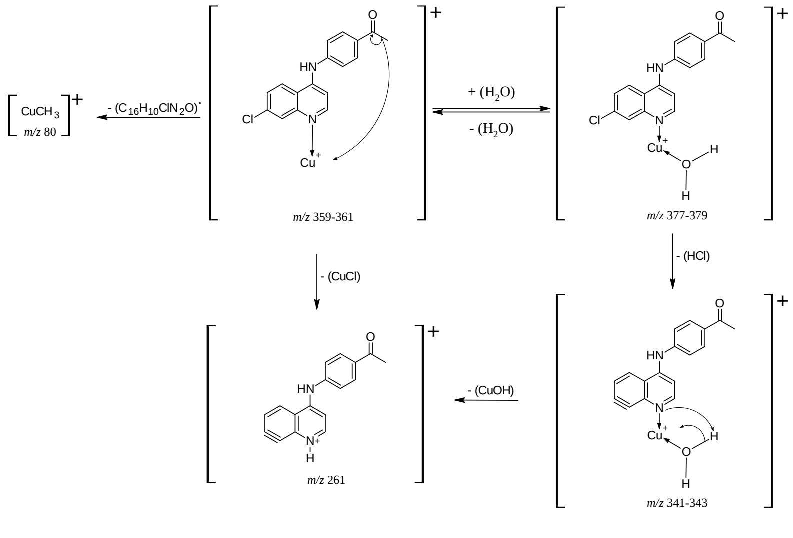

Supplementary Figure 19: Homolytic dissociation and reversible re-coordination/de-coordination of a water molecule for the fragment at *m/z* 359-361, derived from the ion [**4a**]$^+$ at *m/z* 657
